# From Pixel Understanding to Semantic Insight: Intelligent Detection in Sensor-Driven Perception Systems

**DOI:** 10.3390/s26103075

**Published:** 2026-05-13

**Authors:** Qingchen Xie, Tongxu Wu, Fan Yang

**Affiliations:** Department of Automation, Tsinghua University, Beijing 100084, China; xqc23@mails.tsinghua.edu.cn (Q.X.); wutx23@mails.tsinghua.edu.cn (T.W.)

**Keywords:** sensor-driven intelligent detection, multimodal sensing, deep learning, foundation models, multimodal sensor intelligence, fault diagnosis, remaining useful life prediction, non-destructive testing, structural health monitoring, trustworthy deployment

## Abstract

**Highlights:**

**What are the main findings?**

- Intelligent detection is reframed as a sensor-driven state inference problem rather than a purely image-centered recognition task.
- A unified review framework is established through three coupled dimensions (methods, systems, and governance) and four recurring engineering components: signal unification, representation unification, alignment mechanisms, and robustness mechanisms.

**What are the implications of the main findings?**

- The field is shown to be evolving from task-specific models toward integrated sensor-enabled perception systems that couple sensing, computation, decision support, and deployment constraints.
- Future progress depends less on isolated benchmark gains than on mechanism-aware modeling, trustworthy evaluation, missing-modality robustness, privacy-preserving collaboration, and edge-native lifecycle-aware deployment.

**Abstract:**

Intelligent detection in modern manufacturing, healthcare, process industries, and structural monitoring is fundamentally enabled by heterogeneous sensor systems. Rather than being viewed as a purely image-centered recognition task, intelligent detection is more appropriately formulated as a sensor-driven state inference problem in which sensing physics, signal quality, temporal synchronization, modality availability, and deployment conditions jointly determine what can be reliably detected, localized, interpreted, and acted upon. Against this background, this review provides a structured synthesis of the field through three coupled dimensions, namely methods, systems, and governance, and organizes the literature around four recurring engineering components: signal unification, representation unification, alignment mechanisms, and robustness mechanisms. Using a structured review protocol with explicit source selection, screening, and study coding, the paper traces the methodological evolution from traditional feature-engineering and model-based pipelines to deep learning for visual, temporal, multimodal, generative, and mechanism-constrained sensing, and further to foundation-model-based and multimodal sensor intelligence. Cross-domain evidence is synthesized from industrial defect detection, fault diagnosis, remaining useful life prediction, non-destructive testing, structural health monitoring, medical lesion analysis, and process monitoring. The review argues that recent progress has substantially strengthened learned representations, multimodal interaction, and semantic extensibility, but has not removed persistent constraints arising from domain shift, missing modalities, calibration instability, privacy-preserving collaboration, and edge-side resource limits. Accordingly, the central challenge is no longer how to optimize isolated detection models, but how to build sensor-enabled intelligent systems that remain physically grounded, trustworthy, transferable, and maintainable under real operational conditions. On this basis, the paper concludes by identifying future directions in mechanism-aware modeling, trustworthy evaluation, missing-modality-robust multimodal systems, privacy-preserving cross-site collaboration, and edge-native lifecycle-aware deployment.

## 1. Introduction

Intelligent detection refers to the computational identification, localization, characterization, and interpretation of abnormal states, target objects, or clinically and industrially relevant events from sensor-acquired observations [1]. It has become a foundational capability in intelligent manufacturing, condition monitoring, failure prognosis, non-destructive testing, biomedical diagnosis, transportation systems, and structural health monitoring. Importantly, the observational basis of these tasks is inherently multimodal sensor-enabled perception [2,3]. Depending on the application, relevant evidence may be conveyed by RGB images, vibration responses, acoustic emissions, infrared thermograms, hyperspectral or spectral signatures, force/current/voltage measurements, and process variables. For this reason, intelligent detection should not be regarded as a purely image-centric recognition problem. Rather, it is a sensor-driven perception problem in which the quality, structure, and physical meaning of measurements fundamentally constrain what a learning system can detect, explain, and reliably deploy [4].

From this perspective, the main challenge is not limited to algorithmic accuracy. Different sensor types exhibit different measurement physics, noise characteristics, temporal sampling regimes, spatial resolutions, calibration requirements, sensor placement constraints, and failure modes. In practical systems, heterogeneous sensing often introduces asynchronous acquisition, scale mismatch, cross-device variability, missing modalities, and non-stationary drift caused by environmental fluctuations, hardware aging, material changes, or workflow variation. These properties are not peripheral implementation details; they directly shape observability, feature extractability, multimodal alignment, uncertainty, latency, and ultimately the operational reliability of the detection system. Consequently, performance evaluation cannot be reduced to benchmark accuracy alone [5]. It must also account for whether a method remains stable under sensor noise, domain shift, partial observations, and resource-constrained inference at the device, edge, or cloud level.

The development of intelligent detection can therefore be interpreted as a sequence of paradigm shifts driven by both sensing complexity and modeling advances. Early approaches relied on handcrafted descriptors and shallow classifiers, explicitly encoding domain priors through signal processing and feature engineering. Such pipelines often remained effective in small-sample or strongly structured settings, especially when sensing conditions were well understood, but their transferability deteriorated under complex variability and cross-scenario deployment. Deep learning changed this landscape by replacing much of the manual feature design with hierarchical end-to-end representation learning from raw or weakly processed sensor signals. Convolutional, recurrent, attention-based, and generative architectures subsequently expanded the field from visual defect detection to time-series diagnosis, anomaly modeling, and multimodal fusion. More recently, foundation models and multimodal large language models have further extended the scope of intelligent detection from task-specific classification and localization toward shared representation spaces, open-vocabulary perception, explanation generation, and decision support [6]. At a conceptual level, this evolution reflects a transition from explicit prior encoding to implicit representation learning, from single-task mapping to unified cross-modal representations, and from static recognition to knowledge-enhanced reasoning.

Despite this rapid progress, the existing review literature remains fragmented in several important ways. First, many surveys focus on a single application domain—such as industrial surface defect detection [7], rolling bearing fault diagnosis [8], or dermatological image analysis—without examining how common sensing constraints recur across domains. Second, the literature remains strongly biased toward visual detection, while non-visual modalities are frequently treated as auxiliary examples rather than as first-class sources of information with distinct sampling, noise, calibration, and interpretive characteristics. Third, many reviews remain method-centric: they summarize convolutional networks, Transformers, generative models, or large models in parallel, but do not sufficiently explain which classes of methods address which sensor-driven problems, under what assumptions, and at what engineering cost [9]. As a result, central questions for sensor-enabled intelligent detection are rarely synthesized within a common analytical framework. These questions include signal standardization, cross-modal representation compatibility, alignment under asynchronous measurements, tolerance to missing modalities, calibration drift, trustworthy evaluation, and deployability.

This gap is particularly important because intelligent detection is increasingly expected to function as part of an integrated sensing system rather than as an isolated algorithmic module. In modern industrial and biomedical environments, the relevant pipeline includes sensing, calibration, data acquisition, transmission, synchronization, preprocessing, representation learning, inference, human interpretation, and closed-loop action. The effectiveness of a detection method is therefore co-determined by sensing conditions, model architecture, training strategy, system integration, and governance requirements. A review that foregrounds only algorithmic novelty, while backgrounding sensing constraints and operational conditions, cannot fully explain why a method succeeds in one environment yet fails in another. There is thus a clear need for a sensor-centric review that connects methodological evolution to the physical and system-level realities of intelligent detection.

To address this need, this paper reviews intelligent detection from three coupled dimensions: methods, systems, and governance. Rather than cataloguing models in chronological or architectural order alone, we analyze the field through four recurring engineering components that arise across sensing modalities and application domains. The first is signal unification, which concerns denoising, normalization, calibration, resampling, and the transformation of heterogeneous measurements into comparable analytical objects. The second is representation unification, which concerns the learning of shared, transferable, or interoperable latent spaces across tasks, domains, and modalities. The third is alignment mechanisms, which concern temporal, spatial, semantic, and cross-modal correspondence under asynchronous sampling, varying resolutions, and partial observability. The fourth is robustness mechanisms, which concern resilience to noise, drift, domain shift, missing modalities, uncertainty, privacy constraints, and computational limitations. This framework is intended not only to organize the literature more coherently, but also to clarify the functional role and practical limits of different methodological families.

On this basis, the present review covers representative developments spanning traditional feature-engineering pipelines, deep learning architectures for visual and temporal sensing, generative models for anomaly modeling and data augmentation, multimodal fusion methods, vision–language alignment approaches, time-series foundation models, and physics-informed or mechanism-constrained models. The discussion is grounded in application scenarios that are central to sensor-enabled perception, including industrial defect detection, rotating machinery fault diagnosis and failure prognosis, medical skin lesion analysis, multimodal condition monitoring, and related inspection, NDT [10], and structural health monitoring tasks. Instead of emphasizing only the performance gains reported by individual studies, we examine how different methods respond to sensing heterogeneity, incomplete observations, cross-domain deployment, and real-time operational constraints. In this sense, the review is designed to move from “what models exist” to “what sensing and system problems these models solve, and where their limitations remain”.

To improve transparency and reproducibility, the literature considered in this review is collected and screened using a structured review protocol, with the databases, search strategy, inclusion criteria, exclusion criteria, and study classification process described in Section 2. This methodological layer is particularly important because intelligent detection now spans a large and rapidly expanding body of work across computer vision, signal processing, machine condition monitoring, medical artificial intelligence, multimodal learning, and foundation models. A reproducible screening and categorization procedure is therefore necessary to distinguish canonical works, recent representative advances, and application-oriented engineering studies.

The main contributions of this review are as follows. First, it reframes intelligent detection as a sensor-driven systems problem rather than a purely algorithmic recognition task. Second, it proposes a unified analytical framework linking methodological evolution to sensing constraints, system integration, and governance requirements. Third, it offers a problem-oriented synthesis of recent large-model developments by distinguishing advances in representation unification, mechanism-aware modeling, multimodal alignment, missing-modality handling, and deployment efficiency. Fourth, it identifies open research challenges at the interfaces of sensing, learning, and operation, including cross-domain generalization, trustworthy evaluation, privacy-preserving collaboration, and edge-ready adaptation. The remainder of this paper is organized as follows. Section 2 describes the review methodology and the proposed analytical framework. Section 3 formulates the sensor-driven problem space of intelligent detection. Section 4, Section 5 and Section 6 review methodological evolution from traditional pipelines to deep learning and foundation-model-based approaches. Section 7 discusses systems-level evaluation and governance issues, with emphasis on benchmark validity, uncertainty, privacy-preserving collaboration, edge deployment, and lifecycle maintenance. Section 8 synthesizes the comparative strengths, limitations, and future directions of major methodological families across application domains. Section 9 concludes the paper by summarizing the main findings and their implications for trustworthy and maintainable sensor-driven intelligent detection systems.

## 2. Review Methodology and Analytical Framework

### 2.1. Review Objectives and Research Questions

This review was designed as a structured, PRISMA-informed evidence synthesis rather than as a purely architecture-centered narrative survey [11]. The objective was not only to summarize the chronological evolution of intelligent detection methods, but also to explain how sensing constraints, learning paradigms, system integration requirements, and governance considerations jointly shape the development of intelligent detection across industrial, biomedical, and general perception scenarios. This design is motivated by application contexts in which intelligent detection is integrated into end-to-end sensing, monitoring, and decision-support workflows, such as fault diagnosis, failure prognosis, non-destructive testing, sensor fusion, structural health monitoring, digital-twin-assisted analysis, and edge-deployed intelligent sensing [12].

To guide the review, four research questions were formulated. First, what sensing-related constraints recur across intelligent detection tasks, despite differences in applications and modalities? Second, how have methodological paradigms evolved from explicit feature engineering and model-based priors to deep representation learning and foundation-model-based unification? Third, which classes of methods primarily address signal unification, representation unification, alignment, and robustness under realistic sensing conditions? Fourth, what gaps remain when intelligent detection is evaluated not only as an algorithmic task, but also as a deployable sensor-enabled system involving trustworthiness, privacy, cross-domain generalization, and operational sustainability? Framing the review in this way allows the discussion to move beyond a model catalogue and toward a problem-oriented synthesis more suitable for sensor-driven perception systems.

### 2.2. Information Sources and Search Strategy

The review process followed a two-stage identification strategy. In Stage I, a seed bibliography consisting of 81 references was audited to identify topical imbalance, bibliographic inconsistency, and underrepresented research directions. In Stage II, targeted supplementary retrieval was conducted to strengthen the coverage of domains that were insufficiently represented in the seed set, particularly remaining useful life prediction, source-free domain adaptation, federated diagnosis and prognostics, missing-modality learning, time-series foundation models, industrial foundation models, and trustworthy deployment. This second stage yielded 70 additional candidate studies, forming an expanded audit pool for structured screening and synthesis. The use of this two-stage strategy was methodologically necessary because the initial corpus showed uneven topical coverage across sensing modalities, application scenarios, and methodological themes. A structured supplementation stage was therefore required to improve cross-domain balance and to support a more comprehensive sensor-driven synthesis.

Five sources were used to support the identification process: Web of Science Core Collection, Scopus, IEEE Xplore, PubMed, and ScienceDirect. Web of Science Core Collection and Scopus were used as the principal discovery layers because both provide broad cross-disciplinary coverage of journals, conference proceedings, and scholarly books. IEEE Xplore was included as a domain-specific engineering source for IEEE periodicals, conference publications, books, and standards, which are particularly relevant to signal processing, machine condition monitoring, industrial AI, and computer vision. PubMed was used to capture biomedical and clinical strands of the literature, especially for skin lesion analysis, medical imaging, and trustworthy clinical AI. ScienceDirect was used not as a neutral citation index equivalent to WoS or Scopus, but as a complementary full-text and publisher-hosted retrieval layer for engineering and biomedical journals disseminated through Elsevier’s platform.

The primary search window covered January 2018 to March 2026. This range was chosen to capture the maturation of deep learning-based intelligent detection, the rapid expansion of multimodal and foundation-model-based methods, and the recent rise of source-free transfer, federated collaboration, and missing-modality learning. Earlier seminal studies were retained through backward citation chaining when necessary to preserve foundational methodological landmarks, such as canonical feature-engineering pipelines, core object detection architectures, variational and adversarial generative models, and classical representation-learning papers. Forward citation tracking was additionally used for fast-evolving subtopics such as open-vocabulary detection, time-series foundation models, and industrial multimodal reasoning.

The database queries were organized around four Boolean blocks: task terms, sensing terms, method terms, and systems/governance terms. Representative task terms included “intelligent detection”, “defect detection”, “fault diagnosis”, “condition monitoring”, “anomaly detection”, “remaining useful life”, and “prognostics”. Representative sensing terms included “sensor”, “multimodal”, “multi-sensor”, “vibration”, “acoustic”, “infrared”, “thermal”, “spectral”, “hyperspectral”, and “process variables”. Method terms included “machine learning”, “deep learning”, “transformer”, “diffusion”, “foundation model”, “large language model”, “vision-language”, and “multimodal learning”. Systems and governance terms included “domain adaptation”, “source-free domain adaptation”, “federated learning”, “missing modality”, “uncertainty”, “edge deployment”, “sensor fusion”, and “calibration drift”. A representative master query used in the broad search stage can be summarized as follows:

(“intelligent detection” OR “defect detection” OR “fault diagnosis” OR “condition monitoring” OR “anomaly detection” OR “remaining useful life” OR prognostics)

AND

(sensor* OR multimodal OR “multi-sensor” OR vibration OR acoustic OR infrared OR thermal OR spectral OR hyperspectral OR “process variable*”)

AND

(“machine learning” OR “deep learning” OR transformer* OR diffusion OR “foundation model*” OR “large language model*” OR “vision-language” OR “domain adaptation” OR “federated learning” OR “missing modality”)

Because database syntax differs across platforms, this master formulation was adapted to each source using database-specific field restrictions, truncation rules, and phrase formats. For example, topic-field logic was used in Web of Science, title–abstract–keyword logic was emphasized in Scopus and ScienceDirect, IEEE Xplore searches were tuned toward engineering and conference-oriented terminology, and PubMed queries were restricted primarily to Title/Abstract fields to prevent over-expansion into clinically irrelevant biomedical material.

### 2.3. Eligibility Criteria and Study Selection

Studies were considered eligible if they satisfied four conditions. First, they had to address intelligent detection, diagnosis, anomaly detection, prognostics, or closely related perception tasks grounded in sensor-acquired data. Second, they had to involve one or more sensing modalities relevant to industrial, medical, or general sensor-enabled perception settings, including but not limited to images, vibration signals, acoustic signals, infrared data, spectral data, and process variables. Third, they had to contribute representative methodological, benchmarking, engineering, or review value for cross-domain synthesis. Fourth, they had to provide sufficient bibliographic traceability and methodological detail for structured comparison.

The following categories were excluded: studies unrelated to sensor-based detection or diagnosis; generic machine learning papers without meaningful connection to sensing or diagnostic tasks; duplicated records across platforms; non-English editorial material, news items, and informal web pages; and non-traceable or obviously mismatched citations. A limited number of recent preprints were retained only when they represented field-shaping developments that were highly relevant to the current state of the field but had not yet stabilized in archival journal form. This exception was particularly important for fast-moving topics such as industrial foundation models, open-vocabulary multimodal detection, missing-modality learning, and time-series foundation models. At the same time, bibliographic normalization was applied to correct non-traceable, incomplete, or inconsistently formatted references before the analytical synthesis was finalized.

The selection procedure followed four successive stages: database identification, duplicate removal, title/abstract relevance screening, and full-text eligibility assessment. After these steps, backward and forward citation chaining were used to recover seminal or very recent representative studies that would otherwise be missed by keyword-based retrieval alone. This hybrid process was preferred over a one-pass database query because the field spans multiple disciplinary traditions—computer vision, signal processing, machine diagnostics, reliability engineering, medical AI, multimodal learning, and foundation models—which use overlapping but non-identical terminology. In this context, a rigid keyword-only strategy risks both false negatives and thematic imbalance, whereas a structured search plus citation-chaining strategy better supports a cross-domain sensor-centric review.

### 2.4. Study Coding, Evidence Mapping, and Quality Control

To support structured evidence synthesis, each included study was coded along five axes: application domain, sensor modality, method family, problem focus, and systems/governance relevance. Application domains included industrial defect detection, rotating machinery fault diagnosis, remaining useful life prediction and prognostics, skin lesion analysis and medical imaging, general object detection, and related inspection or structural health monitoring settings. Sensor modalities included image, vibration, acoustic, infrared/thermal, spectral/hyperspectral, multi-sensor fusion, and process-variable time series. Method families included handcrafted feature engineering, shallow machine learning, convolutional and recurrent deep models, generative models, multimodal fusion methods, foundation-model-based approaches, physics-informed models, domain adaptation approaches, federated methods, and missing-modality methods [1]. Problem focus was coded as classification, detection, localization, segmentation, anomaly detection, RUL prediction, open-vocabulary detection, missing-modality robustness, efficient deployment, interpretability, or trustworthy evaluation. Systems/governance relevance captured whether a study explicitly addressed domain shift, uncertainty, privacy, edge deployment, human-in-the-loop use, or benchmark contributions.

Quality control was conducted at the bibliographic and analytical levels. At the bibliographic level, metadata consistency, DOI traceability, venue correctness, and source legitimacy were checked to reduce citation mismatch. At the analytical level, the review did not treat all studies as equivalent evidence. Priority was given to canonical foundational works, high-quality peer-reviewed surveys, benchmark papers, and recent engineering studies that explicitly linked model design to sensing conditions or deployment constraints. This was especially important because the initial seed bibliography contained several structural issues: it was comparatively rich in canonical deep learning and vision-language papers, but weaker in methodology reporting, sensor systems analysis, RUL, source-free transfer, federated collaboration, and missing-modality robustness. The targeted supplementation strategy was therefore used not merely to increase the number of references, but to rebalance the evidence base.

### 2.5. Analytical Framework for Sensor-Driven Intelligent Detection

To avoid a purely model-listing narrative, the reviewed literature was synthesized through three coupled dimensions (methods, systems, and governance) and four recurring engineering components (signal unification, representation unification, alignment mechanisms, and robustness mechanisms). This framework serves as the principal analytical lens for the remainder of the review and is intended to bridge sensing constraints with methodological evolution.

Signal unification refers to the preprocessing and normalization operations needed to transform heterogeneous sensor measurements into analytically comparable objects. This includes denoising, calibration, resampling, synchronization, spectral preprocessing, and time–frequency transformation. In a sensor-driven review, signal unification is foundational because different sensing modalities do not enter the learning pipeline in interchangeable form; they first require modality-aware transformation to preserve physically meaningful information.

Representation unification refers to the learning of shared, transferable, or interoperable latent spaces across devices, domains, tasks, and modalities. In traditional pipelines, this was only weakly achievable through manually designed descriptors and feature concatenation. In deep learning and large-model paradigms, representation unification becomes a central objective, enabling cross-modal fusion, zero-shot transfer, and reusable task backbones. This component is therefore essential for understanding why multimodal pretraining, vision–language alignment, and time-series foundation models matter from a sensor perspective.

Alignment mechanisms refer to the strategies used to establish temporal, spatial, and semantic correspondence across heterogeneous measurements. In intelligent detection, alignment is rarely trivial because different sensors may operate at different sampling rates, resolutions, noise levels, or degrees of observability. Alignment therefore includes not only temporal synchronization and spatial registration, but also positional encoding, modality identifiers, cross-attention routing, semantic grounding, and open-vocabulary conditioning.

Robustness mechanisms refer to the means by which intelligent detection systems tolerate non-ideal conditions, including sensor noise, calibration drift, domain shift, missing modalities, data imbalance, privacy constraints, and computational limits. Robustness is especially important in sensor-enabled systems because deployment environments are dynamic and failure-sensitive. A method that performs well on a benchmark but fails under drift, partial observations, or hardware constraints cannot be considered operationally reliable. By structuring the literature around these four components, the review moves from a descriptive inventory of models to a more explanatory understanding of what each methodological family contributes to sensor-driven intelligent detection.

### 2.6. Corpus Characteristics and Distribution of the Reviewed Literature

A preliminary bibliographic audit of the assembled review corpus was conducted before the final narrative synthesis. The seed set contained 81 references, and the targeted supplementation strategy added 70 candidate studies, yielding an expanded audit pool of 151 records. At the document-type level, 87 of these records were journal, review, or guideline articles; 25 were conference papers; 39 were preprints or canonical non-journal works; and the remaining items were non-standard legacy entries requiring bibliographic normalization or replacement. This profile is important because it shows that the assembled corpus is not dominated solely by archival journal articles. It also contains a substantial proportion of fast-moving foundational or preprint literature, which is common in AI-related fields but requires explicit quality control in review writing.

At the level of source traceability, a venue-based audit suggested that approximately 110 records were likely retrievable through Web of Science Core Collection and 120 through Scopus, indicating that these two sources should serve as the principal bibliographic retrieval layers in the documented search workflow. IEEE Xplore, PubMed, and ScienceDirect provided more specialized coverage of engineering, biomedical, and full-text Elsevier-hosted subsets, respectively. This distribution further indicates that the evidence base is not confined to generic computer vision literature, but instead spans industrial AI, signal processing, medical imaging, reliability engineering, multimodal learning, and foundation models.

The corpus audit also revealed a meaningful structural imbalance in the original seed bibliography. The seed set was comparatively strong in visual detection, canonical deep learning backbones, and general multimodal large-model literature, but relatively weak in remaining useful life prediction, source-free domain adaptation, federated prognostics, missing-modality learning, time-series foundation models, and industrial foundation models. The supplementary retrieval stage was therefore explicitly designed to fill these gaps. This rebalancing is methodologically important because, without it, the review would remain overly image-centric and algorithm-centric, thereby limiting its ability to support a genuinely cross-domain sensor-driven synthesis. By contrast, the expanded corpus better supports the analytical framework proposed in this paper and provides more balanced coverage of sensing systems, diagnosis, prognosis, data fusion, and deployment-oriented intelligence.

Based on this structured review protocol and analytical framework, the next section formulates the sensor-driven problem space of intelligent detection by examining how sensing modality, measurement physics, noise, calibration drift, asynchronous acquisition, missing observations, and deployment constraints shape the design and evaluation of intelligent detection systems.

## 3. Sensor-Driven Problem Formulation

Intelligent detection becomes meaningfully “sensor-driven” only when the sensing process is treated as part of the problem definition rather than as a passive input channel. In practical deployments, the measurable evidence available to a model is shaped by sensing physics, acquisition topology, temporal resolution, calibration conditions, environmental perturbations, and the cost of maintaining reliable data streams over time. Consequently, the design space of intelligent detection must be formulated upstream of model selection. It should begin with the question of what can be observed, at what fidelity, under which disturbances, and with what degree of synchronization and operational continuity. From this perspective, learning performance is inseparable from sensing conditions, and the methodological evolution reviewed later in this paper should be interpreted as a sequence of responses to increasingly difficult measurement, integration, and deployment constraints.

### 3.1. Sensor Modalities and Measurement Characteristics

Sensor-enabled intelligent detection is inherently multimodal because different applications expose different physical manifestations of abnormality. In industrial inspection and non-destructive testing, evidence may appear as surface texture changes, subsurface discontinuities, thickness variations, thermal anomalies, or electromagnetic response [10]. In rotating machinery and structural health monitoring, fault-related information is frequently encoded in vibration signatures, acoustic emissions, strain responses, or guided-wave propagation patterns [13,14]. In medical and biochemical analysis, the relevant signal may be visual, spectral, thermal, or multi-parametric [15]. As a result, intelligent detection cannot be reduced to a generic pattern recognition task on interchangeable data types. Each sensing modality carries a specific measurement mechanism and a distinct relationship between the latent physical state and the recorded signal. Reviews of NDT and SHM consistently emphasize that detectability depends not only on algorithmic processing [3], but also on sensitivity, spatial or temporal resolution, penetration depth, environmental susceptibility, and the geometry of sensing and excitation [10].

These differences produce materially different data structures. Image-based sensing usually offers rich spatial context and strong local texture information, but it is sensitive to illumination, viewpoint, occlusion, surface reflectance, and camera-specific artifact. Vibration and acoustic sensing provide dense temporal streams that can preserve weak fault impulses and dynamic evolution patterns [13,14], but they are often contaminated by mechanical noise, load variation, and non-stationary operating conditions. Infrared and thermal sensing capture heat-transfer-related evidence [3] that may reflect friction, material degradation, or abnormal energy dissipation, yet such signals are strongly affected by emissivity assumptions, ambient conditions, and sensor resolution. Spectral and hyperspectral sensing encode material composition and chemical or structural variation in the frequency domain, but they also introduce high-dimensional measurements whose usefulness depends on preprocessing, wavelength selection, scattering correction, and chemometric interpretation [15]. In process industries, electrical and process-variable streams are usually sampled continuously and are central to soft sensing, condition monitoring, and process optimization, but their interpretation depends on system dynamics, control actions, and the degree of observability of hidden process states [16].

From a formulation standpoint, sensor modalities differ not only in data format, but also in the semantics of reliability. For some modalities, the main challenge lies in fine spatial localization; for others, it lies in transient detection, early weak-signal amplification, or trend-sensitive degradation assessment. In NDT applications, the relevant question may be whether a sensing chain can detect a defect below a given size threshold and distinguish surface from internal anomalies. In SHM, the concern may be whether distributed sensing can preserve sensitivity over long-term operation under environmental variability [8]. In process monitoring, the issue may be whether indirect measurements can support sufficiently accurate inference of latent states [17]. Therefore, the same downstream task label, such as detection or diagnosis, may correspond to very different observability structures across modalities. A sensor-driven review must preserve these distinctions rather than flattening them into a single algorithmic narrative.

A further implication is that multimodal sensing is not simply an additive strategy for improving accuracy [3]. Its primary value lies in complementary observability. Different sensors may capture different projections of the same latent abnormality, with distinct noise profiles, temporal responses, and failure sensitivities. This is why recent reviews on multi-sensor fusion consistently organize the field around data-level, feature-level, and decision-level fusion [3,16,18]. The challenge is not merely to concatenate measurements, but to decide when common sampling grids are meaningful, when modality-specific encoders are necessary, and when independent decisions should be fused instead of raw or latent representations. In other words, multimodal sensing broadens what can be observed, but it also increases the burden of standardization, synchronization, and interpretability [2].

### 3.2. From Sensing Constraints to Learning Challenges

The transition from sensing to learning is not transparent. Measurement constraints are translated into learning challenges through several intermediate mechanisms, including information loss, scale mismatch, asynchronous acquisition, non-stationary perturbation, and incomplete observability. When heterogeneous sensors operate at different sampling rates or under different spatial resolutions, the resulting data streams are not directly comparable. When calibration changes over time, the same physical state may generate different statistical patterns. When environmental variation alters background distributions, a detector may face a shift in both signal quality and class boundaries [19]. Accordingly, the true difficulty of intelligent detection is often determined less by model expressiveness alone than by the fidelity with which sensing-related distortions are represented, normalized, or compensated during learning.

One major consequence is that the conventional independent-and-identically-distributed assumption is often violated in real deployments. Cross-device variability, changing operating conditions, material heterogeneity, and environmental disturbance can all induce domain shift between training and testing phases. This has been clearly documented in cross-domain fault diagnosis, where machine load, rotational speed, sensor placement [14], and acquisition chain differences can substantially degrade generalization [20]. Similar concerns arise in medical image analysis, where variable image quality, acquisition protocol differences, and population heterogeneity affect reliability and calibration [21]. As a result, a model that performs well on a benchmark dataset may still fail under real operational conditions unless it is explicitly designed to handle domain shift, uncertainty, and incomplete prior knowledge about target environments.

A second consequence is the emergence of missing-modality and partial-observation problems as first-class learning settings rather than exceptional cases. In realistic systems, modalities may be absent because of sensor faults, communication loss, occlusion, privacy restrictions, maintenance interruptions, or cost-driven sensing policies. Recent surveys on multimodal learning with missing modality show that this problem is structurally different from ordinary multimodal fusion [3,22]. The learning objective is no longer merely to exploit complementary signals when all channels are available, but to preserve useful inference under incomplete, asymmetric, or dynamically changing modality availability. This distinction is particularly important in industrial and biomedical settings, where the most informative modality may also be the least reliable or the most expensive to maintain continuously. Therefore, robustness to missing modalities should be treated as a core formulation issue and not as an implementation detail.

A third consequence concerns label structure and event rarity. Many sensor-driven detection tasks are naturally long-tailed because severe faults, rare lesions, and critical anomalies occur infrequently, while nominal operation dominates the observed data distribution. In addition, annotation quality is itself mediated by sensing conditions. Weak contrast, ambiguous boundaries, low signal-to-noise ratio, or subsurface uncertainty may lead to labels that are sparse, noisy, or only indirectly validated. Larger and more realistic benchmarks have emphasized scale, multi-view variation, and more practical anomaly settings, which make overfitting to overly curated small datasets less likely. Under such conditions, learning challenges include imbalance, threshold instability, confidence miscalibration, and difficulty in maintaining performance under realistic false-positive constraints.

These sensing-induced learning challenges explain why later methodological developments have moved in several directions simultaneously. Signal processing and feature engineering were originally used to stabilize weak observations under known physical conditions. Deep learning introduced hierarchical feature learning to reduce dependence on manual descriptors, but it did not eliminate the need for alignment, calibration, and robustness. Multimodal and foundation-model-based approaches further expanded the representational space, yet they also amplified concerns related to model scale, deployment cost, incomplete observability, and trustworthy behavior. Thus, the formulation of intelligent detection should not be expressed as a simple sequence of better architectures. It should be expressed as a mapping from sensing constraints to learning requirements, including representation compatibility, cross-domain transfer, uncertainty handling, and resilience to degraded inputs.

### 3.3. Systems-Level Implications: Synchronization, Deployment, Robustness

Once intelligent detection is treated as a sensor-enabled system rather than an isolated model, synchronization becomes a central systems problem. In multimodal settings, the challenge is not only how to fuse information, but also how to ensure that information corresponds to comparable physical states in time and space. Different sensors may observe the same object or process with different latencies, acquisition intervals, fields of view, or preprocessing pipelines. For this reason, synchronization must be understood more broadly than timestamp matching. It includes calibration consistency, sampling-rate reconciliation, cross-modal registration, temporal windowing, and the preservation of causal order between sensing and inference. Reviews of industrial sensing, multi-sensor fusion, and SHM indicate that the quality of synchronization directly influences whether cross-modal information becomes complementary evidence or merely additional noise [2,16,18].

Deployment considerations introduce a second systems layer. In practical inspection, monitoring, and prognostics pipelines, inference is only one component of the end-to-end loop. The overall system also includes sensing, signal conditioning, data transport, memory management, runtime scheduling, visualization, human validation, and response triggering. As model complexity increases, computational and communication costs become increasingly difficult to separate from sensing design. Surveys on efficient deep learning have shown that model efficiency depends on architecture, compression, hardware, and infrastructure choices rather than on parameter count alone [18,23]. Recent work on edge intelligence and near-sensor computing further suggests that moving computation closer to the sensing front end can reduce the cost of data transfer and latency, but this benefit comes with constraints on precision, power, thermal envelope, and update flexibility [14,16].

Robustness is the third systems-level implication, and it extends beyond noise tolerance in the narrow algorithmic sense. In sensor-driven systems, robustness includes resistance to calibration drift, resilience to missing or corrupted channels, graceful degradation under domain shift, and reliable confidence behavior under distributional uncertainty [19]. This is especially important in medical and industrial settings, where incorrect outputs may trigger costly interventions or missed alarms. Reviews of uncertainty quantification in medical image analysis have emphasized that predictive confidence must be interpreted with reference to image quality, data variation, and real-world workflow conditions [18]. Similarly, application-oriented reviews of cross-domain fault diagnosis have stressed that robustness requires invariance to changing operating regimes and explicit strategies for unseen target conditions [13,14,20]. At the systems level, this means that trustworthy deployment requires monitoring, recalibration, fallback logic, and, where necessary, human oversight.

Taken together, synchronization, deployment, and robustness define the systems-level boundary conditions within which intelligent detection must operate. They explain why it is insufficient to organize the field solely by network families or benchmark scores. Methods should instead be assessed according to how they interact with sensing constraints, how they support or hinder system integration, and whether they remain reliable under realistic operational variability [24]. This problem formulation also clarifies the logic of the subsequent sections. Traditional machine learning methods are revisited as early solutions built around explicit signal priors and modality-specific processing. Deep learning methods are then analyzed as approaches that expand representation capacity while partially absorbing manual preprocessing into trainable feature hierarchies. Foundation-model-based and multimodal approaches are finally examined as attempts to unify heterogeneous sensing, improve transferability, and support richer system-level intelligence, while also introducing new trade-offs in efficiency, controllability, and trustworthiness.

## 4. Traditional Machine Learning and Model-Based Detection Under Sensor Constraints

The earliest stage of intelligent detection was shaped not by end-to-end representation learning, but by explicit prior encoding. Before the widespread adoption of deep neural architectures, most detection systems were constructed as modular pipelines in which domain knowledge, signal preprocessing, handcrafted feature extraction, and shallow classification or statistical decision rules were combined sequentially. In sensor-driven applications, this design choice was not merely a consequence of limited computational power. It reflected the fact that sensor measurements were understood as physically structured signals whose diagnostic value had to be revealed through carefully designed transformations. Thus, traditional intelligent detection should be interpreted as a family of methods that attempted to compensate for sensing limitations through explicit analytical structure. These methods were especially relevant when the measurement mechanism was reasonably well understood, labeled data were limited, and interpretability or resource efficiency was operationally important. Their limitations, however, became increasingly visible as sensing heterogeneity, cross-domain variation, and system-level integration demands intensified.

### 4.1. Explicit Prior Encoding and Sensor-Specific Signal Processing

A defining property of traditional intelligent detection is that the transformation from raw measurement to diagnostic evidence is largely specified by the analyst rather than learned automatically. This is why early pipelines were strongly sensor-dependent. In non-destructive testing, for example, the core problem is not simply whether a classifier can separate normal and abnormal samples, but whether a sensing modality has sufficient detectability, sensitivity, and resolution for the defect of interest [10]. Conventional inspection methods such as magnetic particle testing, penetrant testing, eddy-current testing, and ultrasonic testing are therefore rooted in modality-specific physical interactions and signal response mechanisms. Their role in intelligent detection is foundational because they establish the measurable signal geometry on which later data-driven analysis depends. In this setting, explicit prior encoding takes the form of measurement setup design, excitation strategy, defect-oriented signal interpretation, and threshold-based or feature-based decision rules.

The same logic appears in visual inspection, although the measurement space is spatial rather than vibrational or electromagnetic. In classical image-based detection, the raw image is rarely treated as a directly learnable representation. Instead, it is converted into engineered descriptors designed to preserve local shape, gradient, or texture regularities while suppressing nuisance variation. Representative examples include SIFT, HOG [25], and LBP [26], which were designed to encode local geometric salience, oriented gradients, and texture micro-patterns, respectively. In industrial inspection, such descriptors were often paired with image normalization, threshold segmentation, morphology-based preprocessing, or region proposal heuristics before being passed to shallow classifiers such as support vector machines or random forests. The sensor-driven rationale is clear: when image quality is degraded by illumination changes, surface reflectance, background clutter, or limited defect contrast, explicit feature design serves as a mechanism for signal stabilization and dimensionality reduction.

In condition monitoring based on vibration and acoustic signals, explicit prior encoding is even more pronounced because diagnostically relevant information is usually embedded in weak transients, modulated periodicity, or frequency-localized signatures that are not directly separable in the raw time domain. Traditional fault diagnosis therefore relies heavily on signal decomposition and feature engineering. Time-domain statistics, frequency-domain descriptors, envelope spectra, cepstral characteristics, and time-frequency transforms such as short-time Fourier transform, wavelet analysis [17], spectral kurtosis, and envelope demodulation are used to amplify fault-sensitive components before classification. Recent reviews of rotary machine fault diagnosis still present this signal-processing layer as a core methodological foundation [8], not merely as a historical artifact, because it remains effective whenever fault mechanisms are understood and the objective is to detect interpretable fault signatures under limited data conditions. In this sense, the traditional pipeline is not “pre-deep-learning” in a simplistic chronological sense; it is a problem formulation that assumes the existence of physically meaningful intermediate representations.

In spectral sensing and chemometric analysis, the same principle appears in a different mathematical form. Spectroscopic measurements are high-dimensional, strongly correlated, and often influenced by baseline drift, scattering effects, path-length variation, and instrumental noise. Accordingly, the traditional workflow emphasizes signal correction and latent-variable extraction before classification or regression. Standard normal variate, multiplicative scatter correction, derivative preprocessing, principal component analysis, linear discriminant analysis, partial least squares, and support vector machines are widely used because they provide interpretable routes from raw spectra to compact, task-relevant latent variables. Reviews of food spectroscopy repeatedly emphasize that the usefulness of the downstream model is inseparable from appropriate chemometric handling of the sensor signal [15]. This again shows that traditional intelligent detection was fundamentally organized around modality-aware preprocessing and explicit statistical structure.

A related but often underemphasized branch of traditional sensor-enabled detection lies in process monitoring and soft sensing. In process industries, many quality variables of interest cannot be measured directly or continuously, while pressure, temperature, flow rate, current, and other auxiliary signals are available at high frequency. This gave rise to soft sensors and multivariate statistical process monitoring, in which latent variables, correlation structure, and change statistics are used to infer hidden quality states or detect abnormal operation [27]. Classical tools such as PCA, PLS, Hotelling’s $T^{2}$, squared prediction error, CUSUM, and Shewhart-type schemes remain important because they encode process structure, temporal continuity, and control-oriented interpretability. Reviews of industrial sensing and control continue to present hybrid modeling, soft sensing, and statistical monitoring as practically successful approaches precisely because they align with measurement availability, process constraints, and decision-making requirements.

### 4.2. Shallow Learning Pipelines Across Representative Modalities

Although traditional methods are often summarized as “feature extraction plus classifier,” that description is too coarse for a sensor-driven review. In practice, traditional pipelines are heterogeneous because the definition of a useful feature depends on the sensing modality, the target event, and the operational cost of false alarms or missed detections. In image-centered inspection, the classifier often receives manually designed geometric or textural representations [25] whose value lies in discriminability under limited data. In vibration-based diagnosis, the classifier operates on signal features already shaped by assumptions about fault periodicity, resonance amplification, and modulation behavior. In spectral or process monitoring tasks, decision-making is mediated by latent subspace projections and residual statistics rather than by direct discrimination in the raw sensor space [15]. Therefore, shallow learning in intelligent detection should be understood as a downstream inference layer appended to a sensor-specific evidence construction process.

For this reason, many representative traditional methods are better interpreted as hybrid analytical pipelines rather than standalone machine learning algorithms. In anomaly screening with scarce positive samples, one-class support vector machines [28], isolation forests, and local outlier factor methods are often used not in isolation, but together with reconstructed residuals, sparse coding error, or latent subspace deviations [27]. Their main purpose is to define a compact notion of normality in feature space and then measure departures from it. In sensor-driven environments, this is particularly valuable when abnormal states are rare, weakly labeled, or operationally heterogeneous. The strength of such approaches lies in their ability to work with limited supervision and modest computational overhead. Their weakness is that the boundary of “normality” is highly sensitive to how the sensor signal was normalized and represented beforehand. Thus, even anomaly-oriented traditional learning methods remain tightly coupled to signal engineering.

In rolling bearing and machinery diagnosis, shallow classifiers such as support vector machines, k-nearest neighbors, decision trees, random forests, and hidden Markov models historically played an important role because they could exploit compact condition indicators derived from expert-designed signal analysis. Reviews of rolling bearing fault diagnosis show that such methods achieved meaningful performance [8] when fault types were relatively structured, operating regimes were controlled, and the sensing configuration was stable. However, they also reveal a recurrent limitation: the boundary between classes is rarely invariant across sensor position, rotational speed, mechanical load, or environmental disturbance. As a result, the practical burden of traditional learning often shifts from classifier selection to feature redesign, signal denoising, and operating-condition compensation. This is precisely where the later transition to learned representations gains its justification.

In multi-sensor settings, traditional approaches typically adopt one of three forms: early fusion of standardized measurements, feature-level concatenation of sensor-specific descriptors, or decision-level aggregation of independent diagnostic outputs [2]. Even before modern cross-attention or multimodal representation learning, these fusion schemes already reflected a recognition that no single sensing channel provides sufficient observability in many industrial scenarios. Recent reviews on multi-sensor fault diagnosis still classify fusion along these levels, which shows that the basic fusion logic predates deep architectures. What distinguishes the traditional regime is that fusion is usually manually orchestrated. The analyst decides how signals are synchronized, which handcrafted descriptors are comparable, how to weight sensor contributions, and whether residual disagreement should be treated as uncertainty or noise. This manual nature makes such methods interpretable and operationally transparent, but it also limits scalability when modalities become numerous, asynchronous, or only intermittently available.

Overall, traditional intelligent detection methods were strongly shaped by sensing modality, feature construction, and decision strategy. Table 1 summarizes several representative traditional methods and their typical advantages and limitations.

### 4.3. Strengths, Structural Limits, and the Transition Pressure Toward Learned Representations

Traditional machine learning and model-based detection remain important not because they are historically earlier, but because they embody several enduring strengths. First, they are often highly interpretable. The transformation from signal to feature to decision is usually explicit, which is valuable in safety-critical environments where operators need to understand why a system raises an alarm. Second, they can be effective under small-sample conditions because they inject strong priors about the measurement process and reduce the burden of representation learning. Third, they are often computationally efficient and suitable for deployment on constrained hardware or in situations where training data, annotation capacity, or infrastructure support are limited. These strengths explain why explicit feature engineering, statistical process monitoring, and model-based reasoning remain active in inspection, prognosis, and process monitoring workflows even after the rise of deep learning.

However, the structural limitations of this paradigm become increasingly severe as sensing environments become more heterogeneous. The first limitation is weak representation portability. Handcrafted features that are effective for one sensor, one material class, or one operating regime often fail to transfer across devices, domains, or environments. The second limitation is poor support for unstructured multimodal fusion. Traditional fusion strategies can combine a few predefined signals, but they struggle when modalities differ strongly in scale, temporal density, or semantic content. The third limitation is brittleness under drift and partial observability. Because much of the intelligence resides in manually designed preprocessing and feature choices, calibration changes, missing channels, or unanticipated noise patterns can invalidate the assumptions embedded in the pipeline. The fourth limitation is the narrow scope of inference. Traditional pipelines are generally optimized for fixed-label discrimination or statistical deviation detection, not for open-set adaptation, explanation generation, or reusable cross-task representations. These limitations are not incidental engineering annoyances. They are precisely the pressure points that pushed the field toward end-to-end representation learning.

From the perspective of the analytical framework introduced in Section 2, traditional methods are strongest in signal unification and weakest in representation unification. They can normalize, denoise, resample, decompose, and summarize modality-specific signals with considerable effectiveness when the sensing mechanism is known. They can also provide partial robustness mechanisms through thresholding, residual analysis, and change detection. By contrast, they provide only limited support for learning shared latent spaces across modalities, operating regimes, and tasks. Their alignment mechanisms are also largely manual, relying on explicit synchronization rules, handcrafted correspondence, or separately engineered fusion stages rather than jointly optimized alignment. This asymmetry explains both the historical success and the eventual insufficiency of the traditional paradigm. It succeeded because sensor-driven problems initially demanded careful signal handling more than large-scale representation learning. It became insufficient because modern intelligent detection increasingly requires transferability, multimodal interoperability, and system-level adaptability.

Accordingly, the transition to deep learning should not be framed as a simple replacement of weak methods by stronger ones. A more accurate interpretation is that deep architectures emerged to absorb, generalize, and partially automate functions that traditional pipelines handled manually. Convolutional networks reduced dependence on handcrafted visual descriptors. Temporal models and attention mechanisms reduced dependence on fixed time-frequency templates. Generative models reduced dependence on manually specified anomaly criteria in some settings. Yet the sensor-driven issues highlighted in the previous section did not disappear. Rather, they were reformulated at a different level of abstraction. This is why the next section turns to deep learning not as a separate topic disconnected from traditional methods, but as the next methodological response to the same family of sensing constraints.

## 5. Deep Learning for Sensor-Driven Intelligent Detection

The transition from traditional pipelines to deep learning did not eliminate the sensor-driven nature of intelligent detection. Rather, it redefined where prior knowledge and adaptation mechanisms are placed within the analytical chain. In traditional systems, signal conditioning, feature engineering, and decision boundaries were specified explicitly by the analyst. In deep learning systems, a substantial part of this processing burden is transferred into trainable hierarchical representations. This shift is particularly important in sensor-enabled applications because the observed data are often high-dimensional, nonlinear, and strongly affected by noise, scale variation, and cross-domain heterogeneity. Deep learning therefore became attractive not only because it improved benchmark accuracy, but also because it provided a mechanism for learning feature hierarchies directly from images, temporal signals, and multimodal measurements that were difficult to characterize exhaustively through handcrafted rules. At the same time, this transition did not remove the importance of sensing conditions. Instead, it made the interaction between sensing quality, data volume, model architecture, and deployment constraints more tightly coupled.

### 5.1. Deep Spatial Representation Learning for Visual and Image-Centered Sensing

**Figure 1:**
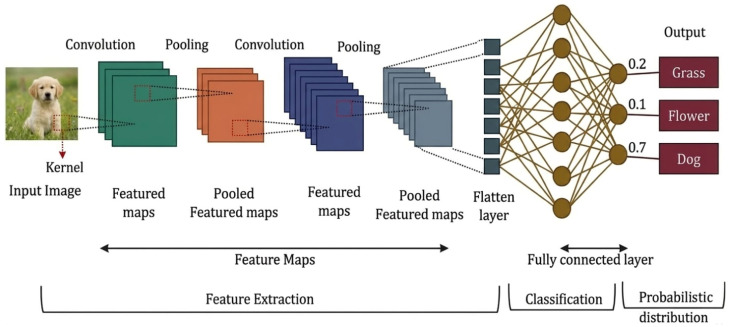
Schematic diagram of a convolutional neural network (CNN) architecture. Created by the authors.

In image-centered sensing tasks, deep learning introduced a major methodological change by replacing manually designed descriptors with hierarchical feature extractors trained in an end-to-end manner. Convolutional neural networks (CNN) became the dominant backbone [29] because their local receptive fields, weight sharing, and compositional depth allowed them to learn progressively richer representations, from low-level gradients and textures to defect morphology, lesion structure, and object semantics [30]. Figure 1 illustrates a generic convolutional feature-extraction pipeline underlying this stage of deep visual representation learning.

In industrial surface inspection, this shift was particularly consequential because defect visibility is often degraded by low contrast, cluttered textures, scale variation, and illumination inconsistency. Recent systematic reviews of industrial surface defect detection show that the most effective deep pipelines [7] are not defined simply by classifier choice, but by how they combine convolutional backbones with multi-scale feature aggregation, localization heads, and training strategies that remain sensitive to subtle and small targets [31].

From a sensor-driven perspective, the significance of CNN-based detectors lies in their ability to internalize part of the feature extraction process that previously had to be hand-designed. Two-stage and one-stage detectors, including Faster R-CNN [32], SSD [33], and the YOLO family [34], can all be interpreted as different compromises between localization precision, computational efficiency, and robustness to background variability. In industrial inspection, feature pyramid structures [35] and deformable operators [36] gained particular importance because surface defects frequently appear at multiple scales and with irregular geometry. Accordingly, the practical value of these architectures is not that they are universally superior, but that they can better preserve fine-grained evidence under real sensing imperfections while maintaining usable throughput for deployment. Reviews focused on industrial object detection repeatedly identify small-defect sensitivity, speed-accuracy trade-offs, and adaptation to complex backgrounds as the central engineering challenges in this domain [37].

Medical image analysis exhibits a related but distinct pattern. Here, deep learning is valuable because medical and dermoscopic images often contain ambiguous boundaries, subtle texture changes, and pronounced inter-patient variation, all of which complicate rule-based segmentation and diagnosis. CNN-based segmentation and classification frameworks therefore became central to skin lesion analysis, lesion boundary delineation, and pixel-level risk localization. Recent surveys and application studies indicate that modern deep systems in dermatological imaging [38] frequently combine preprocessing, classification, and segmentation modules rather than relying on a single prediction stage. This is also visible in recent skin lesion pipelines such as SkinProNet [39], which integrate preprocessing, EfficientNet-derived [40] feature extraction, recurrent attention-enhanced classification, and U^2^-Net-type segmentation modules. The sensor-driven implication is that deep visual models are most effective when they are treated as components of an image-quality-aware sensing workflow rather than as stand-alone classifiers.

However, deep spatial representation learning also introduced new dependencies. Its success is often contingent on annotation quality, imaging consistency, and sufficiently broad training coverage [41]. In both industrial and medical settings, performance may degrade when acquisition devices change, image quality deteriorates, or target morphologies fall outside the training distribution. For this reason, the deep visual stage should not be interpreted as having solved the sensing problem. It solved part of the handcrafted representation problem, but it simultaneously increased sensitivity to dataset bias, confidence miscalibration, and deployment mismatch [19]. These limitations explain why later work increasingly incorporated data augmentation, anomaly modeling, domain adaptation, and uncertainty-aware evaluation rather than relying on supervised image recognition alone.

### 5.2. Deep Temporal and Multimodal Learning for Dynamic Sensor Signals

**Figure 2:**
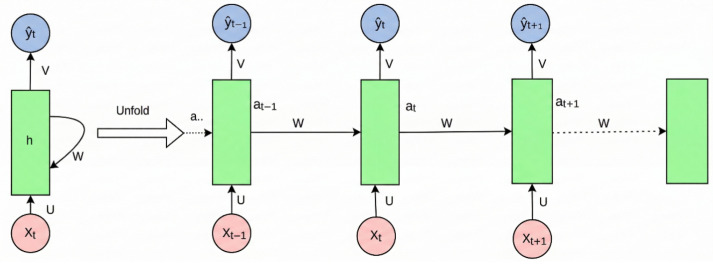
Schematic diagram of the recurrent neural network (RNN) architecture. Reproduced from [44], licensed under CC BY 4.0.

The impact of deep learning was equally significant in temporal sensing, especially in fault diagnosis, condition monitoring, and process-variable analysis. Unlike visual inspection, these tasks depend on the recovery of dynamic patterns from vibration, acoustic, thermal, electrical, or process streams, where fault information may be distributed across long temporal windows or localized in transient events. In this context, one-dimensional convolutional networks, recurrent neural networks, long short-term memory networks, gated recurrent units [42], temporal attention modules, and hybrid CNN-sequence architectures became influential because they could model temporal structure directly [43] from raw or weakly processed signals. The basic recurrent computation pattern underlying early deep temporal modeling is illustrated in Figure 2. Its relevance in this review lies in showing how deep learning extended intelligent detection from static visual evidence to temporally evolving sensor streams.

Recent critical reviews of rolling bearing fault classification [8,20] emphasize that deep temporal models improved fault recognition by reducing reliance on rigid handcrafted descriptors while still benefiting from sensor-aware preprocessing when signals were noisy or operating conditions changed [13,45,46]. Reviews of machine learning in vibration and acoustics similarly show that deep learning expanded the range of detectable phenomena but did not eliminate the importance of denoising, windowing, spectral transformation, or the physical interpretation of measured dynamics [13].

This point is particularly important for rotating machinery, acoustic monitoring, and thermal condition assessment. In these settings, deep learning does not simply replace signal processing; it often builds on it. Time-frequency maps, spectrograms, wavelet coefficients [17], envelope spectra, or fused modality-specific features are frequently used as inputs to deep networks because they preserve diagnostically salient structure that raw sequences alone may not expose efficiently. Accordingly, the practical advantage of deep temporal models lies in their capacity to learn higher-order correlations, nonlinear interactions, and temporal dependencies after the sensor signal has been rendered into a more learnable form [45,46]. This is one reason why hybrid architectures remain common [47]. They combine sensor-aware preprocessing with trainable feature extraction and are therefore better suited to fault signatures that are weak, non-stationary, or distributed across multiple scales.

Deep learning also expanded the feasibility of multimodal sensor fusion before the emergence of foundation models. In rotating machine diagnosis and related monitoring problems, recent reviews consistently classify deep fusion into data-level, feature-level, and decision-level integration [2], but unlike traditional fusion pipelines, deep architectures can learn nonlinear interdependencies between modalities through shared encoders, dual-branch networks, attention-based interaction modules [48], or modality-specific subnetworks followed by fusion heads [49]. This is especially useful when vibration, acoustic, infrared, and process-variable signals offer complementary but imperfect observability of the same underlying fault state. At the same time, these methods exposed a recurring systems difficulty already outlined in Section 3. Asynchronous sampling, inconsistent resolution, missing modalities, and variable signal quality limit how much fusion can be learned reliably from data alone. Thus, deep multimodal learning improved cross-sensor evidence aggregation, but it also made the need for alignment and robustness mechanisms more explicit [50].

A related development occurred in process industries and soft sensing. As large-scale industrial systems generated increasingly rich streams of process variables, deep feedforward, recurrent, and autoencoder-style architectures [43] began to be used for quality prediction, virtual sensing, and nonlinear process-state inference. Surveys on industrial sensing and control show that deep learning attracted attention in soft sensing because it could capture nonlinear relationships that were difficult to model with classical latent-variable methods alone [51]. However, these same surveys also emphasize that interpretability, data efficiency, and operational trust remain central concerns, which is why hybrid modeling and process-aware design continue to be recommended [45,46]. In sensor-driven terms, deep soft sensing is not merely another prediction task. It is an attempt to infer latent process states from incomplete and indirect measurements, which makes the quality of sensing, historical coverage, and operating-regime diversity critical to model validity.

### 5.3. Deep Generative Modeling for Anomaly Representation, Augmentation, and Reconstruction

A major extension of deep learning in intelligent detection came from generative modeling. While discriminative architectures are optimized to classify, localize, or segment known categories, generative models address a different but equally important problem: how to characterize normality, synthesize scarce data, or reconstruct expected signal structure under weak supervision. This shift is highly relevant in sensor-driven applications because severe faults, subtle defects, and rare lesions are often underrepresented, weakly annotated, or difficult to define exhaustively in closed-set labels. Industrial anomaly detection surveys therefore treat deep generative methods as a central methodological branch [52] rather than a peripheral enhancement. Their main value lies in learning data structure beyond explicit label boundaries [45,46], which makes them useful for anomaly localization, small-sample augmentation, and semi-supervised analysis [53].

**Figure 3:**
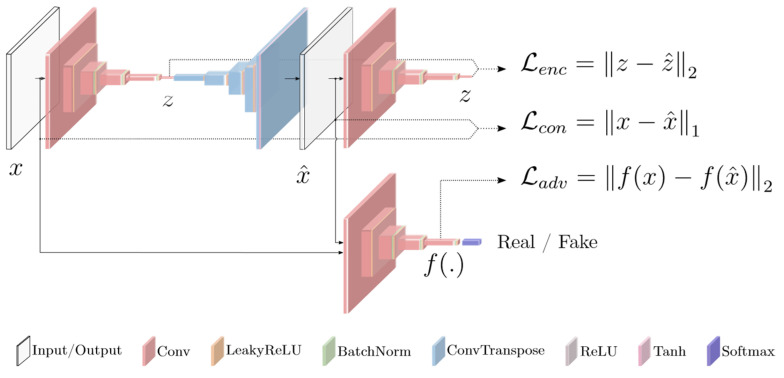
General architecture of GANomaly. Reproduced from [54], with permission from Springer Nature.

Generative adversarial networks and variational autoencoders were particularly influential in this phase. Representative anomaly-oriented adversarial frameworks, such as GANomaly [54], AnoGAN [55] and F-AnoGAN [56], further illustrate how encoder–decoder consistency can be used to model normality and localize deviations. One representative anomaly-oriented generative framework is shown in Figure 3.

GAN-based methods enabled the synthesis of defect-like or lesion-like samples to mitigate imbalance [57,58,59], but they also supported anomaly modeling through reconstruction inconsistency between observed inputs and learned normal manifolds. Variational autoencoders [60] provided a probabilistic alternative, allowing anomaly evidence to be interpreted through latent distribution mismatch and reconstruction residuals. In industrial settings, these approaches were especially attractive when labeled abnormal samples were scarce and when the goal was to detect departures from a learned notion of normal operation or appearance [61]. In medical imaging [62], similar mechanisms were used to support uncertainty-aware reconstruction and to complement discriminative segmentation systems under limited-sample conditions [63]. The broader significance of this phase is that deep learning began to move beyond direct recognition toward latent distribution modeling [64], which widened the methodological role of deep architectures in sensor-driven detection.

**Figure 4:**
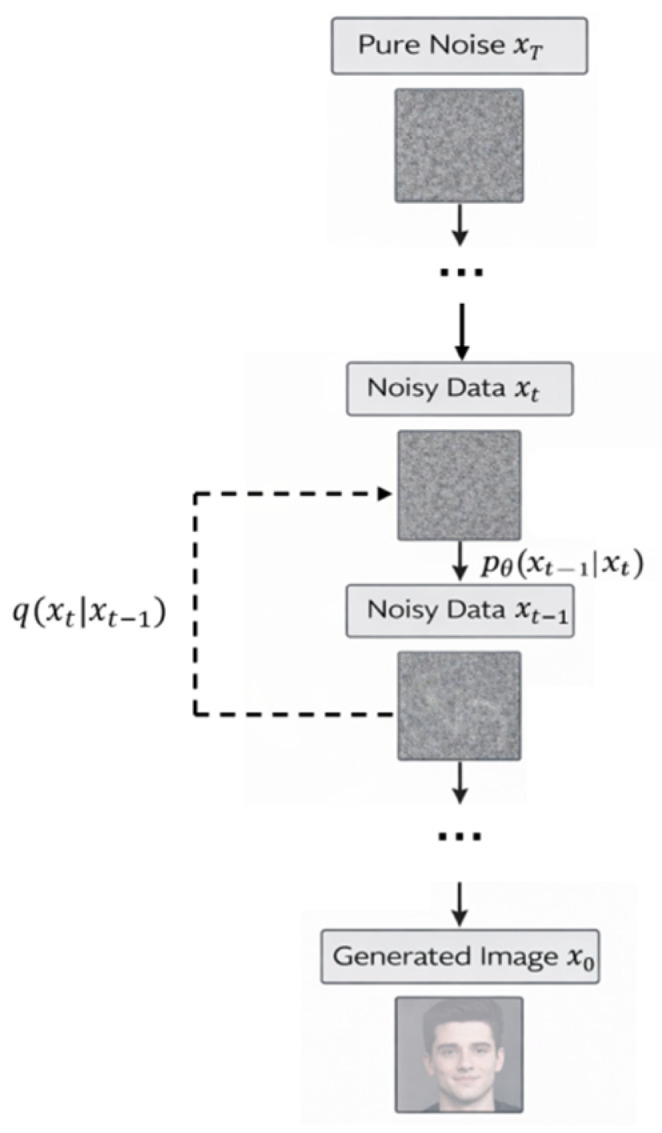
Schematic illustration of the denoising process in diffusion models. Adapted from [65].

Diffusion models [65] extended this trajectory by offering stronger generative fidelity and more stable training behavior, albeit often at greater computational cost. Recent work in industrial anomaly detection has highlighted diffusion-based pipelines [66] as promising because they can reconstruct anomaly-free counterparts [67] or model normal-state structure through iterative denoising. The forward noising and reverse denoising logic of diffusion-based modeling is illustrated in Figure 4.

This iterative denoising principle is central to understanding why diffusion models became relevant for anomaly reconstruction and structured inference. At the same time, diffusion mechanisms have also been adapted to direct detection formulations, such as viewing object detection as a box-denoising process [68]. This broadening of the generative paradigm is important because it shows that deep learning in intelligent detection was no longer limited to supervised recognition or latent reconstruction. It began to support iterative inference, structured uncertainty [69], and more flexible formulations of abnormality. However, recent reviews also emphasize that inference efficiency, deployment feasibility, and robustness under realistic conditions remain major constraints for diffusion-based methods.

From a sensor perspective, the importance of generative modeling is twofold. First, it provides a way to handle sparse labels and rare events by shifting part of the problem from category discrimination to structure recovery or normality estimation. Second, it partially decouples detection performance from exhaustive annotation, which is valuable in industrial inspection, weak-fault diagnosis, and certain medical screening scenarios. Its limitations, however, are equally significant. Synthetic fidelity does not guarantee operational realism, reconstruction quality does not always translate into reliable anomaly localization, and computational burden may conflict with real-time deployment. As a result, generative models should be viewed as complementary mechanisms within deep intelligent detection systems rather than universal replacements for discriminative learning.

### 5.4. Physics-Informed and Mechanism-Constrained Deep Learning

**Figure 5:**
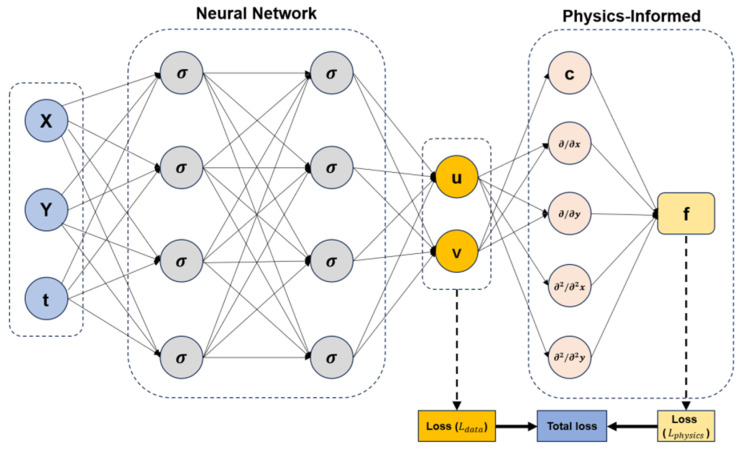
Overall architecture and loss formulation of physics-informed neural networks (PINNs). Adapted from [73], licensed under CC BY 4.0.

While the deep learning methods discussed above substantially improved visual representation learning, temporal pattern extraction, and anomaly modeling, they remained predominantly data-driven in their optimization logic. In many sensor-enabled applications, however, reliable inference depends not only on fitting observed data, but also on maintaining consistency with governing physics, process mechanisms, and measurement constraints. This requirement is particularly important in fault diagnosis, prognostics, non-destructive testing, structural health monitoring, and soft sensing, where sensor observations are often indirect, noisy, incomplete, or weakly labeled. In such settings, purely correlation-driven models may produce predictions that are statistically plausible but physically inadmissible. This challenge motivated the emergence of physics-informed and mechanism-constrained deep learning as an important branch of the deep learning stage [70]. Physics-informed neural networks [71] are the most representative formulation of this branch, because they incorporate differential equations, boundary conditions, conservation laws, or constitutive relations into network training and thereby constrain learning within a physically admissible solution space [51,72]. This view is consistent with recent studies and reviews, which increasingly treat PINNs as a representative framework [73] for integrating observational data and physical knowledge within learning-based inference [74]. A simplified formulation of this idea can be written as follows:

(1) $$L\left(\theta \right)=L_{data}+\lambda_{p}L_{physics}+\lambda_{b}L_{bc}+\lambda_{i}L_{ic}$$

where $L_{data}$ denotes the discrepancy between model predictions and sensor observations, $L_{physics}$ represents the residuals of the governing equations, and $L_{bc}$ and $L_{ic}$ enforce boundary and initial conditions, respectively. In this formulation, learning is no longer guided solely by observational fit. Instead, the admissible hypothesis space is further restricted by physical consistency, which is precisely why PINNs are attractive in sensor-driven settings characterized by sparse measurements, incomplete observability, and mechanism-sensitive inference. The architecture and loss decomposition of a representative physics-informed neural network are illustrated in Figure 5. Placed here, the figure helps clarify that mechanism-constrained deep learning is defined not by model size, but by the explicit incorporation of physical consistency into the learning objective.

From a sensor-driven perspective, the main value of PINNs lies in their ability to connect measured signals with latent physical states more tightly than conventional deep models. In condition monitoring and prognostics, many target quantities of interest, such as degradation states, hidden process variables, stress evolution, or thermal transport patterns, are not observed directly and must instead be inferred from sparse sensor streams under uncertainty. Physics-informed learning is especially valuable in such settings because it can reduce ambiguity in inverse inference, improve stability under limited supervision, and enhance plausibility under out-of-distribution conditions. Recent reviews in prognostics, health management, and condition monitoring likewise emphasize that physics-informed machine learning is particularly useful when data are scarce, extrapolation is unavoidable, or hidden-state inference must remain consistent with process knowledge. Recent work on remaining useful life prediction further illustrates this tendency. For example, an interpretable serialized variational autoencoder has recently been proposed from a drift-diffusion stochastic equation perspective for RUL estimation, which suggests that latent degradation modeling in deep prognostics is increasingly being coupled with more explicitly structured and interpretable dynamic assumptions. This development is particularly relevant to sensor-driven intelligent detection because it shows that deep generative representations can be made more informative for prognostic inference when the evolution of latent health states is constrained by degradation-oriented process structure rather than learned as a purely unconstrained statistical embedding.

This line of work also extends beyond classical PINNs toward operator-learning methods such as DeepONet [75] and Fourier Neural Operator [76]. These methods are relevant because they learn mappings between functions or fields rather than only pointwise input-output relations, which is valuable when intelligent detection must reason about temperature evolution, stress distribution, flow behavior, degradation trajectories, or simulation-to-measurement transfer. Recent reviews describe neural operators as an important framework for learning operators on continuous domains and for supporting efficient field-level inference, including settings that require extrapolation to new locations or resolutions. In the context of intelligent detection, they are most useful when simulation consistency, latent-state reconstruction, or multi-resolution physical reasoning must be combined with sensor data interpretation.

At the same time, the contribution of mechanism-constrained deep learning should be stated with precision. These methods do not eliminate the need for high-quality sensing, and they do not automatically solve multimodal alignment, cross-device calibration, or semantic transfer. Their effectiveness depends strongly on the validity of the embedded physical prior, and PINN training can be sensitive to loss balancing, sampling strategy, and optimization stiffness. Neural operators likewise offer strong expressive power, but their generalization still depends on the structure of the target operator and the adequacy of the training distribution. Therefore, physics-informed and mechanism-constrained deep learning should not be portrayed as a universal substitute for either classical physics-based modeling or general-purpose deep learning. Its real significance lies in providing a principled middle ground between them [70], especially in sensor-driven scenarios where measurement and mechanism are tightly coupled.

### 5.5. Critical Synthesis of Deep Learning in Sensor-Driven Intelligent Detection

Compared with the traditional paradigm discussed in Section 4, deep learning substantially expanded the methodological scope of intelligent detection. It reduced dependence on handcrafted descriptors in visual sensing, strengthened temporal and multimodal pattern extraction in machinery and process monitoring, introduced generative strategies for anomaly representation and scarce-data regimes, and further diversified into physics-informed and mechanism-constrained learning for physically admissible inference. Taken together, these developments transformed intelligent detection from a pipeline centered on manual evidence construction into a broader family of trainable systems capable of learning from heterogeneous sensor observations at multiple levels of abstraction.

When assessed against the four-component analytical framework introduced in Section 2.5, the achievements of this stage are significant but uneven. Deep learning improved representation unification more than traditional pipelines because hierarchical encoders could absorb part of the modality-specific preprocessing burden into trainable feature hierarchies. It also strengthened alignment mechanisms to a limited extent through shared encoders, attention modules, and learned multimodal fusion. At the same time, physics-informed and mechanism-constrained models demonstrated that deep learning could also respond to mechanism constraints, thereby making inference more physically grounded in selected sensor-driven scenarios. However, the contribution of this stage to signal unification remained conditional, since calibration, denoising, synchronization, and modality-aware preprocessing were still often required upstream of learning, especially in temporal, spectral, and industrial sensing environments. Its contribution to robustness mechanisms was likewise incomplete. Domain shift, missing modalities, asynchronous fusion, confidence miscalibration, annotation sparsity, and deployment overhead continued to constrain practical reliability, particularly in medical and industrial settings where performance must remain stable under changing sensing conditions.

Accordingly, the deep learning stage should be understood as a major expansion rather than a final resolution of sensor-driven intelligent detection. It made learned representations more expressive, more adaptive, and in some cases more physically grounded than those of traditional pipelines, yet most models remained task-specific, modality-specific, and dataset-specific. They were typically optimized for fixed supervision regimes, fixed sensor combinations, and fixed operating contexts, and therefore did not yet provide the reusable, cross-task, and semantically extensible representation space that later became central to foundation-model-based approaches. In this sense, the significance of the deep learning stage lies not only in the performance gains it achieved, but also in the methodological tensions it exposed. These tensions include the growing dependence on large annotated datasets, the incomplete handling of missing and asynchronous modalities, the persistent gap between semantic success and operational trustworthiness, and the difficulty of achieving broad transfer under heterogeneous sensing conditions. Seen in this light, deep learning should be regarded as the stage that greatly broadened the representational, generative, and mechanism-aware capacity of intelligent detection, while still leaving unresolved the problem of how to achieve scalable, reusable, and broadly transferable sensor intelligence.

## 6. Foundation Models and Multimodal Sensor Intelligence

The transition from task-specific deep learning to foundation-model-based approaches marks a further change in how representation learning is organized in intelligent detection. Whereas deep learning primarily reduced reliance on hand-crafted features via end-to-end hierarchical encoders, foundation models aim to reduce the dependence on task-specific training itself. Its defining characteristic is not model scale alone, but the use of large-scale pretraining, reusable latent representations, semantically extensible interfaces, and lightweight downstream adaptation to support transfer across tasks, modalities, and application settings. In recent surveys, this shift is commonly described as a movement from specialist architectures toward more general-purpose multimodal systems. In sensor-driven intelligent detection, this development is especially important because heterogeneous sensing environments demand models that can operate across different measurement forms, supervision regimes, and task definitions more flexibly than earlier deep architectures allowed.

At the same time, foundation-model-based sensor intelligence should not be understood as a purely semantic or scale-driven extension of conventional deep learning. Its practical relevance depends on whether broad pretrained representations can remain compatible with the physical structure, quality variation, and incompleteness of real sensor data. In other words, these models become meaningful in intelligent detection only when representation reuse is balanced against sensing-aware requirements such as calibration consistency, cross-modal alignment, missing-modality tolerance, uncertainty control, and efficient adaptation under domain-specific constraints. This is particularly important in industrial and medical environments, where measurements are often heterogeneous, partially observed, weakly labeled, and operationally sensitive. For this reason, foundation models are best interpreted here not as universal replacements for prior sensor-aware methods, but as a new methodological layer that substantially strengthens representation unification and semantic transfer while still depending on signal unification, alignment, and robustness mechanisms established earlier in this review.

### 6.1. Representation Unification Through Large-Scale Multimodal Pretraining

A central motivation for foundation models in sensor-enabled intelligent detection is the need for reusable representations that are not narrowly tied to a single task or sensing configuration. This need was only partially addressed by conventional deep learning [5,77,78]. Task-specific CNNs, temporal models, and fusion networks learned useful internal features, but their latent spaces were typically optimized for fixed modality combinations and narrowly defined supervision [79,80]. By contrast, foundation-model-based pretraining aims to construct broader representational spaces that can support transfer across heterogeneous data types, output formats, and downstream tasks [5,6,81]. This is the conceptual background behind the growing importance of general multimodal backbones and universal perceptual encoders.

Perceiver IO [82] is a representative example of this shift. Its architectural premise builds on the latent bottleneck design introduced by Perceiver [83]. Its importance lies less in a specific application benchmark than in its architectural premise that arbitrary structured inputs and outputs can be handled within a common latent-query framework, while retaining favorable scaling with respect to input and output size. From a sensor perspective, such an architecture is attractive because heterogeneous measurements often differ sharply in length, resolution, and semantic granularity. A common latent interface makes it easier to conceptualize sensing problems as query-driven latent representations rather than as collections of modality-specific networks. Likewise, DINOv2 [84] illustrates the value of large-scale pretraining for producing transferable visual features that remain useful across image-level and pixel-level tasks [84,85] with limited or no fine-tuning. Although DINOv2 is not itself a multimodal model, it helps define the broader foundation-model regime by showing that sufficiently curated large-scale pretraining can produce robust, reusable perceptual representations beyond narrowly supervised pipelines.

ImageBind [86] extends this logic into explicitly multimodal space by learning a shared embedding across images, text, audio, depth, thermal signals, and inertial measurements, using image-paired data as the binding mechanism. This is especially relevant to sensor-driven intelligent detection because thermal and IMU-like signals already belong to the operational sensing vocabulary of industrial monitoring and mobile perception. The methodological significance of ImageBind is not simply that it supports more modalities, but that it provides a concrete demonstration of representation unification without requiring full co-occurrence of all modalities during training. In principle, this offers a route for linking partially overlapping sensing sources into a common latent space, which is highly attractive in industrial settings where synchronized multimodal corpora are often incomplete or expensive to construct. However, the usefulness of such unification still depends on whether semantically aligned embeddings preserve the fault-sensitive or safety-critical structure that downstream decisions require.

From the standpoint of the analytical framework proposed in this review, large-scale multimodal pretraining primarily strengthens representation unification. It improves the possibility of shared embeddings, cross-modal transfer, and reusable backbones across tasks. What it does not solve automatically is signal unification. Sensor calibration, denoising, resampling, spectral transformation, and quality control remain necessary whenever the physical meaning of the measurements is fragile or modality-specific. This distinction is important because it prevents foundation models from being misrepresented as a universal substitute for sensing-aware preprocessing. In many real systems, especially those involving vibration, thermal, spectral, or process-variable data, the value of a unified latent space still depends on the quality and consistency of the measured signal entering that space.

### 6.2. Language-Guided Perception and Open-Vocabulary Sensor Intelligence

**Figure 6:**
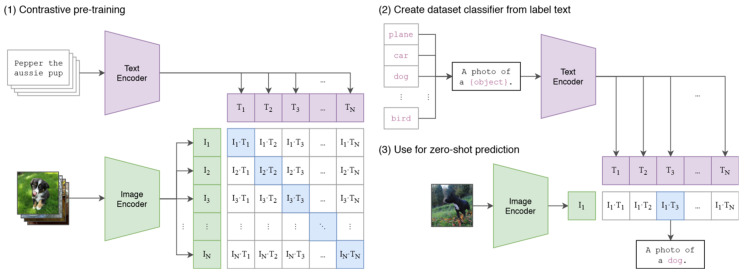
Architecture of CLIP for pretraining and inference. Reproduced from [87], licensed under CC BY 4.0.

A second major development in the foundation-model stage is the introduction of language as an operational interface for perception [9]. CLIP [87] was pivotal in this transition because it demonstrated that large-scale contrastive training on image-text pairs could produce visual representations that transfer zero-shot to many downstream tasks. Figure 6 illustrates the contrastive image–text pretraining paradigm underlying language-guided perception.

For intelligent detection, the importance of this result is not limited to image classification. It shows that category definitions can be mediated by natural language rather than by a fixed closed-set label space. This is especially valuable in long-tail inspection and diagnosis scenarios, where annotated categories are incomplete, evolving, or expensive to define exhaustively in advance.

This language-guided paradigm subsequently expanded into open-vocabulary detection. OWL-ViT [88] showed that contrastively pretrained image-text models can be adapted to end-to-end open-vocabulary detection. GLIP [89] further unified object detection and phrase grounding during pretraining, thereby learning object-level, language-aware representations with strong zero-shot and few-shot transfer potential. Grounding DINO [90] advanced this direction by combining detector design with grounded pretraining and cross-modal fusion, allowing arbitrary textual prompts or referring expressions to guide open-set detection. In methodological terms, these models represent an important transition from category-specific detectors to semantically extensible perception systems. In sensor-driven intelligent detection, this matters because defect taxonomies, maintenance events, symptom descriptions, and clinician notes are often expressed linguistically before they are formalized as rigid labels. A language-conditioned perception model therefore offers a more natural interface between sensor observations and expert knowledge.

**Figure 7:**
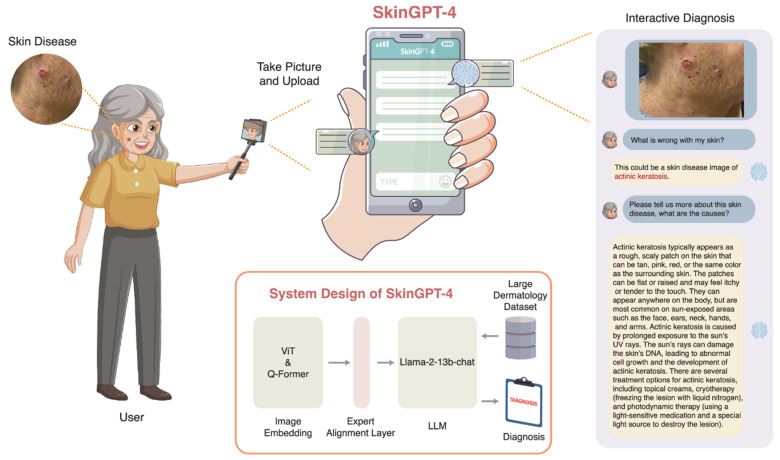
Detection architecture and application examples of SkinGPT-4. Reproduced from [21], licensed under CC BY 4.0.

The same trend is visible in biomedical applications. SkinGPT-4 [21] is a particularly relevant example because it demonstrates that multimodal large language models can align visual encoders with large language backbones to support not only diagnostic prediction, but also interactive explanation and report-style reasoning in dermatological contexts. A representative medical multimodal language-model pipeline is shown in Figure 7.

For this review, the significance of such systems is not simply that they are multimodal, but that they connect perception outputs with textual interpretation and clinically meaningful communication. This extends intelligent detection from recognition toward explanation and decision support, which is highly consistent with the sensor-system perspective established earlier in the paper. Nevertheless, this extension also introduces new concerns, including semantic overgeneralization, prompt sensitivity, hallucination risk, and uncertain calibration when language priors dominate weak visual evidence. These risks are particularly consequential in medical and high-stakes industrial environments, where plausible-sounding explanations are not equivalent to trustworthy evidence.

Accordingly, language-guided sensor intelligence should be interpreted as a strengthening of semantic alignment mechanisms, not as a replacement for physically grounded detection. It is most useful when the system must bridge sensor observations with open-vocabulary concepts, diagnostic text, maintenance logs, operator instructions, or downstream reporting. It is less reliable when the critical challenge lies in fine-grained physical discrimination that has weak linguistic supervision or limited representation in pretraining corpora. Therefore, the value of vision-language and multimodal language models in intelligent detection is real, but conditional. Their greatest contribution lies in task extensibility, human-interpretable interfaces, and rapid semantic adaptation, whereas their weakest point remains the controlled handling of rare, subtle, and physically constrained abnormal patterns.

### 6.3. Cross-Modal Alignment and Missing-Modality Robustness in Sensor Fusion

If representation unification and language guidance are two pillars of the foundation-model stage, the third is robust multimodal alignment under incomplete sensing. This issue is central to sensor-driven intelligent detection because real systems rarely provide all modalities at equal quality and at all times. Sensors may be unavailable because of hardware failure, communication loss, maintenance interruptions, occlusion, privacy restrictions, or cost-driven acquisition policies. Recent surveys on deep multimodal learning with missing modality explicitly distinguish this setting from ordinary multimodal fusion [22]. The problem is no longer simply how to combine multiple signals when they are all present [2]. Instead, the objective is to preserve reliable inference when modality availability changes dynamically across training and deployment.

This challenge has motivated a shift from static fusion toward representation designs that separate shared and modality-specific information, or that use prompts and auxiliary structures to compensate for absent channels. ShaSpec is a representative example because it learns shared and specific features jointly and uses auxiliary alignment mechanisms to maintain performance under missing modalities [91]. More recent prompt-based multimodal methods have extended this idea by introducing generative prompts, missing-signal prompts, or missing-type prompts, thereby treating absent modalities as structured conditions rather than as simple data corruption [92,93,94]. For a sensor-driven review, these developments are highly relevant because they point toward robustness mechanisms that are compatible with heterogeneous acquisition and partial observability [95]. They also reinforce the view that missing modalities are not an exception at the edge of deployment, but a design condition that should shape the architecture of intelligent detection systems from the outset.

In industrial settings, the practical meaning of missing-modality robustness is even broader [96]. It encompasses intermittent thermal channels, unavailable auxiliary process variables, irregular vibration coverage, partial camera views, and cross-device sensing inconsistencies. It also includes situations where the most informative sensing channel is too expensive, too fragile, or too privacy-sensitive to operate continuously. In such environments, robust multimodal models must do more than interpolate missing inputs. They must preserve task-relevant evidence while controlling uncertainty under incomplete observation. This is one reason why modality-aware weighting, prompt-conditioned inference, and shared-latent designs are becoming more important than naive feature concatenation. Yet this progress should not be overstated. Learned cross-modal alignment is not equivalent to physically valid synchronization, and embedding-level compatibility does not guarantee sensor-level consistency. The systems issues identified in Section 3 therefore remain fully relevant in the foundation-model stage.

Emerging application studies in the sensor-enabled fault diagnosis literature illustrate both the promise and the current incompleteness of this direction. Recent work has shown that multimodal large models can be fine-tuned for machine fault diagnosis using non-contact dynamic vision data from event cameras [24], thereby extending diagnostic pipelines beyond traditional attached vibration sensing. Such studies are important because they indicate that large multimodal models can begin to absorb novel sensor types into the diagnostic workflow. At the same time, they also highlight a central limitation of the present stage. These systems remain highly application-specific, and their performance still depends on carefully constructed domain datasets, adaptation protocols, and sensing conditions. In other words, the move toward multimodal sensor intelligence has clearly begun, but it has not eliminated the need for domain-specific alignment and validation.

### 6.4. Temporal Foundation Models, Process Signals, and Industrial Foundation Models

Many sensor-enabled intelligent detection systems are organized around continuously acquired temporal measurements, including process variables, equipment telemetry, thermal histories, electrical signals, and degradation trajectories. In such settings, the relevance of time-series foundation models does not lie merely in generic forecasting capability. More importantly, these models provide reusable temporal priors that can support long-horizon representation learning [74] over sensor streams whose structure is difficult to capture through task-specific supervision alone. This is why temporal foundation models are increasingly relevant to remaining useful life estimation, prognostics, soft sensing, early anomaly screening, and multimodal industrial monitoring [79,80].

Models such as TimeGPT [97] and TimesFM were originally developed as general-purpose foundation models for time-series forecasting and demonstrated strong zero-shot or few-shot transfer across diverse temporal datasets [98]. Their direct contribution to sensor-driven intelligent detection should therefore be interpreted with precision. They are not yet universal fault diagnosis models in themselves. Rather, they offer pretrained temporal backbones that can encode long-range dependencies, regime variation, and multiscale temporal regularities in sensor-generated sequences. In industrial settings, this makes them potentially valuable as temporal experts within multimodal systems, where process variables, load histories, or degradation-related signals must be integrated with visual, acoustic, or thermal evidence. At the same time, their current strengths remain more mature in forecasting [97] than in fault-sensitive representation learning, which means that domain-specific adaptation and evaluation are still indispensable when these models are transferred to diagnosis, prognostics, or anomaly detection tasks. This limitation is especially important in industrial environments, where temporal sensor streams rarely exist in isolation and must often be interpreted together with visual inspection data, thermal measurements, maintenance records, and process semantics.

For this reason, recent research has increasingly moved beyond standalone temporal pretraining [77,99,100] toward broader industrial foundation models that aim to integrate temporal, perceptual, and operational information within a unified adaptation framework [5,77,78,101]. Recent surveys define industrial foundation models as models trained or adapted for industrial data and workflows [77], with intended applicability to predictive maintenance, fault diagnosis, quality inspection, process intelligence, and human–machine collaboration [5,78]. Closely related surveys on large-scale foundation models for intelligent manufacturing and on foundation models for the process industry make a similar point from adjacent perspectives. The industrial setting differs from generic internet-scale multimodal pretraining because the underlying data are often sparse, proprietary, highly heterogeneous, and strongly shaped by process knowledge, engineering documents, maintenance records, and device-specific operating conditions. Accordingly, industrial foundation models are not merely domain-specialized descendants of general models. They are responses to the fact that industrial intelligence requires joint treatment of sensing, process semantics, decision support, and deployment constraints.

**Figure 8:**
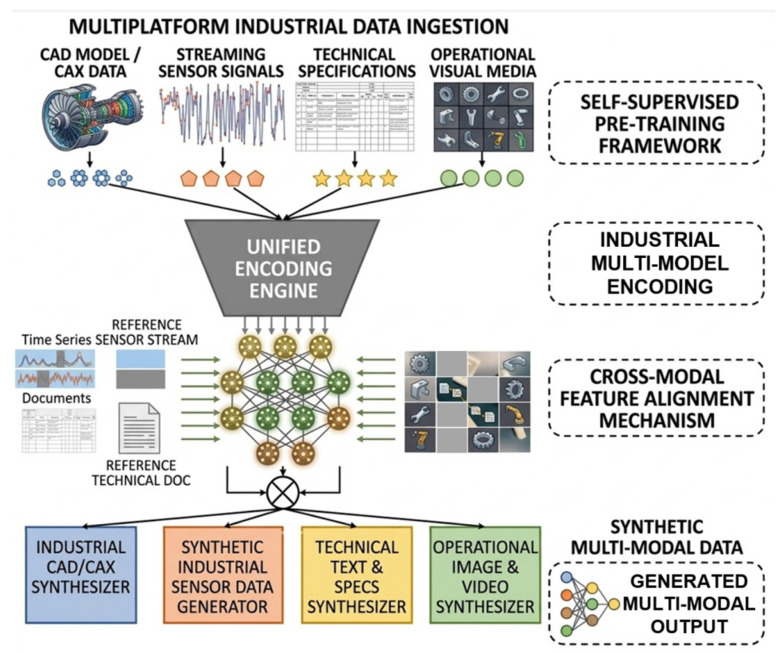
Intelligent detection based on multimodal sensor data fusion with industrial foundation models. Adapted from [77].

From a sensor-driven standpoint, the significance of industrial foundation models lies in their attempt to unify evidence streams that have historically been modeled separately, including machine vision, vibration and thermal signals, process variables, inspection text, maintenance logs, and operator instructions. Figure 8 summarizes a representative industrial foundation-model workflow for integrating heterogeneous sensing streams into a unified perception and decision-support pipeline [100]. This is precisely the systems-level integration logic that distinguishes industrial foundation models from narrower task-specific multimodal detectors.

This is precisely the form of cross-modal interoperability that the analytical framework in Section 2 and Section 3 identified as increasingly necessary. At the same time, their present limitations should be stated with equal clarity. Industrial foundation models must operate under fragmented data governance, limited benchmark realism, confidentiality constraints, shifting equipment populations, and the requirement that transferred representations remain physically plausible under changing operating conditions. Therefore, although industrial foundation models represent a strategically important direction for intelligent detection, their current maturity should be described with precision. They are emerging as an organizing framework for sensor-enabled industrial intelligence, but their practical strength still depends heavily on domain-specific data curation, adaptation, and evaluation.

### 6.5. Efficient Adaptation and Deployment Constraints for Foundation Models

The foundation-model stage also changes how adaptation and mechanistic knowledge are incorporated into intelligent detection. Because model size and pretraining cost continue to grow, full fine-tuning becomes increasingly impractical in many industrial and medical settings. This is why parameter-efficient adaptation methods such as LoRA [102] are important in the current stage. LoRA freezes the pretrained backbone and introduces low-rank trainable updates, thereby reducing trainable parameter count and memory cost while preserving competitive downstream performance. In sensor-enabled intelligent detection, such methods are valuable because domain adaptation often has to be performed under limited compute budgets, restricted data access, and rapidly changing task definitions [23]. Their role is therefore not auxiliary. They are part of the practical infrastructure that makes large models deployable in specialized sensing environments.

**Figure 9:**
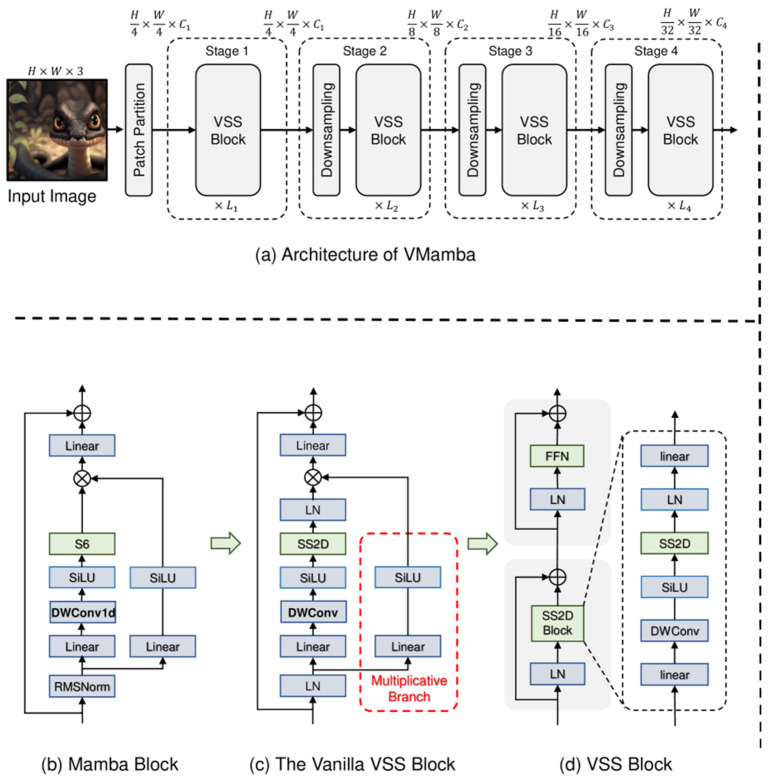
Overall architecture of VMamba and the design of its Mamba/VSS modules. Reproduced from [108], with permission from [108].

Efficiency at sequence scale is another reason that newer backbone families matter [103,104,105,106]. Mamba and related selective state-space models [107] are relevant because they aim to preserve strong sequence modeling capability while scaling linearly with sequence length, which is attractive for long-horizon monitoring, high-frequency sensing, and edge-constrained deployment [23]. A representative state-space backbone for efficient long-sequence modeling is illustrated in Figure 9.

Its relevance in the present section lies in showing why efficient sequence backbones matter for deployment-aware foundation-model adaptation rather than for architectural novelty alone. This does not make such backbones universal replacements for Transformers, but it does signal an important methodological point. In sensor-driven applications, the value of a foundation model cannot be judged only by semantic flexibility. It must also be judged by whether it can operate under the latency, memory, and throughput constraints of real sensing pipelines. This makes efficient sequence backbones, compression-aware adaptation, and hardware-conscious design intrinsic to the foundation-model discussion rather than secondary engineering details.

### 6.6. Critical Synthesis of Foundation-Model-Based Sensor Intelligence

Relative to the deep learning stage discussed in Section 5, foundation-model-based sensor intelligence introduces a qualitatively different form of methodological generalization. Its main contribution is not merely larger model scale, but the construction of reusable multimodal backbones, semantically extensible interfaces, and broader transfer across sensing modalities, task formulations, and supervision regimes. In this sense, the present stage expands intelligent detection from task-specific feature learning toward more general representation spaces that can be conditioned by language, adapted through prompts or lightweight updates, and reused across heterogeneous sensing environments. This is why open-vocabulary detection, multimodal grounding, time-series pretraining, and industrial foundation models are increasingly treated as parts of a shared emerging landscape rather than as isolated technical threads.

At the same time, these advances remain uneven when evaluated through the analytical framework proposed in Section 2.5. Foundation models are strongest in representation unification, because they substantially improve the possibility of shared latent spaces, transferable backbones, and semantic interaction across modalities. Their contribution to alignment mechanisms is also meaningful, but still conditional, since cross-modal correspondence learned during large-scale pretraining does not automatically guarantee physically valid synchronization, modality consistency, or fault-relevant alignment in real sensing systems. By contrast, their contribution to signal unification remains limited, because calibration, denoising, resampling, and measurement-quality control still depend on modality-aware preprocessing. Their contribution to robustness mechanisms is likewise incomplete. Missing modalities, synchronization errors, hallucination, confidence miscalibration, domain shift, and deployment overhead continue to constrain reliability in practical settings, particularly in industrial and medical environments where semantic plausibility is not equivalent to trustworthy inference.

Accordingly, the foundation-model stage is best understood as a powerful but incomplete response to the sensor-driven challenges identified earlier in this review. It substantially broadens the representational and semantic capacity of intelligent detection systems, yet it does not eliminate the need for sensing-aware preprocessing, domain-specific validation, uncertainty control, and deployment-oriented evaluation. In other words, foundation-model-based sensor intelligence should not be interpreted as the endpoint of methodological evolution, but as a transitional stage in which stronger transferability and richer semantic interfaces are achieved faster than fully reliable operational intelligence. This tension between representational generality and sensing-grounded reliability remains one of the central issues that future research must address.

## 7. Systems, Evaluation, and Governance

The preceding sections traced the methodological evolution of intelligent detection from traditional feature-engineering pipelines to deep learning and foundation-model-based sensor intelligence. However, methodological sophistication alone is not sufficient for judging whether a detection system is practically reliable [1]. In sensor-enabled environments, the performance of intelligent detection depends not only on representational capacity, but also on the validity of the data used for development, the realism of benchmark assumptions, the stability of inference under uncertainty, the possibility of privacy-preserving collaboration, and the feasibility of deployment under strict latency and resource constraints. As a result, the latter stage of a sensor-driven review must move beyond architecture-level comparison and address systems, evaluation, and governance as first-class analytical dimensions. This shift is consistent with recent literature across industrial anomaly detection, medical AI, federated diagnosis, and edge intelligence, all of which increasingly emphasize that operational credibility depends on more than benchmark accuracy or model novelty.

### 7.1. Data Quality, Benchmark Bias, and Cross-Domain Validity

The first systems-level issue concerns the relationship between data quality and evidential validity. In sensor-driven intelligent detection, performance is strongly mediated by the physical and operational properties of the data collection process. Image quality may vary with illumination, exposure, optics, and viewpoint. Vibration and acoustic signals may shift under load variation, installation differences, background interference, and sensor aging [109]. Infrared and spectral measurements may be affected by emissivity assumptions, ambient conditions, scattering, and calibration quality. Accordingly, the training and evaluation data used in intelligent detection are never neutral carriers of task information. They are shaped by sensing conditions that may or may not match the environments in which the system will eventually operate. This is why the quality of a benchmark cannot be evaluated solely by its scale, annotation density, or popularity. It must also be evaluated by its fidelity to realistic sensing conditions, its coverage of operational variation, and its capacity to expose rather than conceal domain shift.

This issue has become increasingly visible in industrial anomaly detection and cross-domain fault diagnosis [7,20,110]. Newer real-world industrial datasets such as AeBAD, Real-IAD, and ISP-AD were proposed precisely because earlier benchmarks tended to assume favorable imaging conditions, limited domain diversity, or insufficiently realistic deployment variation [52,111,112]. These newer datasets emphasize domain shift, multi-view diversity, imperfect imaging conditions, and real production variability, thereby exposing the gap between benchmark success and real operational generalization [37]. Likewise, recent application-oriented reviews of domain generalization for fault diagnosis show that cross-device, cross-load, cross-speed, and cross-environment variability remains a defining challenge in rotating machinery and related monitoring tasks [19,113]. Together, these developments indicate that cross-domain validity should be treated as a primary evaluation criterion rather than as a secondary robustness check [114,115,116,117,118,119,120]. A benchmark that does not meaningfully probe domain shift, calibration variation, or sensor inconsistency risks overstating the maturity of the underlying methods [121].

From the standpoint of review structure, this means that the discussion of datasets and evaluation must move beyond score reporting. The important questions are whether the benchmark reflects the sensing regime of interest, whether training and testing distributions are unrealistically aligned, whether annotations capture operationally meaningful targets, and whether the reported metrics are sensitive to long-tailed events, false-positive tolerance, and out-of-distribution degradation. In industrial settings, this implies that multi-view variation, illumination change, background contamination, device heterogeneity, and class rarity should be represented more explicitly. In medical settings, it implies that acquisition protocol differences, patient heterogeneity, and image-quality variation should be incorporated into evaluation rather than treated as nuisance. In process and prognostics settings, it implies that temporal non-stationarity, regime switching, and incomplete observability should be reflected in both dataset design and benchmarking protocols [24]. Cross-domain validity is therefore not an optional extension of model evaluation, but a central requirement for sensor-enabled intelligent detection.

### 7.2. Trustworthiness, Uncertainty, Calibration, and Auditability

The second major issue concerns the trustworthiness of model outputs. In sensor-driven intelligent detection, a prediction is rarely valuable in isolation. Its operational meaning depends on whether its confidence is calibrated, whether its errors are monitorable, whether uncertainty is visible to downstream users, and whether the system can be audited after deployment [122]. This is especially important in industrial maintenance and medical diagnosis, where false alarms, missed detections, and overconfident errors may trigger costly interventions or unsafe inaction [21]. Recent reviews of uncertainty quantification in medical image analysis repeatedly emphasize that predictive uncertainty must be interpreted relative to data quality, distributional variability, and workflow context rather than as a purely statistical appendage to a classifier [123]. The same logic applies to industrial systems [124], where decision thresholds often interact with maintenance cost, production tolerance, and risk asymmetry.

Calibration therefore deserves a more central role in intelligent detection than it has often received. A model may be accurate on average yet poorly calibrated under rare faults, degraded sensor conditions, or unseen domains. Under such circumstances, even well-performing detectors may become unreliable components in a larger sensing system because their confidence scores no longer correspond to actual empirical risk. This suggests that evaluation protocols should routinely incorporate calibration error, confidence reliability, abstention or rejection behavior, and uncertainty-stratified performance analysis. A commonly used calibration measure is the expected calibration error, defined as follows:

(2) $$ECE=\sum_{m-1}^{M}\;\frac{\left|B_{m}\right|}{n}\left|acc\left(B_{m}\right)-conf\left(B_{m}\right)\right|$$

where $B_{m}$ denotes the set of predictions whose confidence falls into the *m*-th bin, $|B_{m}|$ is the number of samples in that bin, n is the total number of evaluated samples, $acc(B_{m})$ is the empirical accuracy within the bin, and $conf(B_{m})$ is the mean predicted confidence. Lower ECE indicates closer agreement between confidence and empirical correctness [125]. In sensor-driven intelligent detection, this quantity is particularly informative when models are evaluated under domain shift, degraded sensing quality, rare-event conditions, or incomplete observations, because it makes visible whether apparently strong predictive performance is accompanied by reliable confidence behavior [62].

In medical AI, this trend is already visible in recent trustworthy AI discussions that combine discrimination, calibration, uncertainty quantification, and clinical workflow relevance [126,127]. A similar shift is needed in sensor-driven industrial review writing, where performance tables should be supplemented with analyses of threshold stability, uncertainty under drift, and behavior under incomplete or low-quality measurements.

Auditability is the corresponding governance layer. If a model is deployed in a sensor-enabled system, it should be possible to reconstruct what data regime it was trained on, what evaluation assumptions were made, what calibration conditions were verified, and what limitations were declared. Recent analyses of model cards show that documentation practices are now widespread but uneven in informativeness, while newer work on model reporting and audit cards argues that evaluation documentation should include scope, methodology, access conditions, review process, and trustworthiness context rather than merely task labels and scores. For intelligent detection, this implies that future systems should be accompanied not only by dataset names and accuracy figures, but also by statements about sensing conditions, domain coverage, confidence calibration, known failure modes, and audit trails for deployment updates [23]. In other words, trustworthiness in sensor-driven detection requires both uncertainty-aware modeling and structured reporting.

### 7.3. Privacy-Preserving and Federated Collaboration

The third major issue concerns collaborative learning under privacy and data-governance constraints. Sensor-driven intelligent detection increasingly depends on geographically distributed and institutionally fragmented data sources. In medical imaging, patient data often cannot be centralized because of privacy regulation and institutional control. In industrial monitoring, sensor data are frequently proprietary, site-specific, and operationally sensitive. In multi-factory or multi-client scenarios, centralized training may therefore be infeasible even when collaboration would clearly improve generalization. Federated learning has emerged as one of the most visible responses to this problem [128] because it enables shared model training without requiring the transfer of raw local data. Recent reviews in predictive maintenance, medical imaging, and intelligent fault diagnosis [129,130,131,132] consistently describe federated and privacy-preserving learning as strategically important directions [133,134] for turning isolated local models into more transferable collaborative systems [135,136].

However, privacy-preserving collaboration cannot be reduced to a simple replacement of centralized aggregation with federated optimization [137]. The deeper difficulty is that local clients often differ substantially in sensing conditions, class distributions, domain coverage, and operational priorities. In industrial prognostics and health management, one site may collect high-quality vibration data under stable loads, whereas another may operate under different sensor placement, maintenance schedules, degradation patterns, and noise conditions. In medical imaging, institutions may likewise differ in hardware vendors, acquisition protocols, patient populations, and annotation conventions [138]. Under such statistical and sensing heterogeneity, a single global model may become suboptimal or even unreliable if client-specific structure is ignored. This is why recent research has moved beyond simple global averaging toward personalized federated learning, federated transfer learning, federated meta-learning, and uncertainty-aware federated strategies [139]. The same tendency is also evident in cross-domain remaining useful life prediction, where recent source-free domain adaptation work from a distributed federated learning perspective [140] has shown that privacy preservation and domain adaptation are not separate concerns, but coupled requirements in heterogeneous sensing environments. The practical implication is that collaborative intelligence in sensor-driven systems must jointly balance privacy preservation, heterogeneity management, client adaptation, and reliability under uneven sensing conditions.

A sensor-centric review should therefore frame federated learning not merely as a privacy technology, but as a governance and systems technology. Its relevance lies in enabling cross-site knowledge accumulation without raw data pooling, while also exposing new questions about communication cost, parameter consistency, local calibration, uncertainty sharing, and accountability across participants. Future work in this direction should pay particular attention to three issues. First, privacy should be evaluated jointly with utility and robustness, because privacy-preserving mechanisms may degrade rare-event sensitivity or calibration. Second, heterogeneity-aware collaboration should be prioritized over naive consensus, especially in fault diagnosis and medical imaging where sensor and domain differences are structural rather than incidental [141,142,143,144]. Third, documentation and auditability should extend to collaborative training itself, including what was aggregated, under what protocol, and with what client diversity. In this sense, federated collaboration belongs in a systems and governance chapter rather than in a generic training-strategy chapter.

### 7.4. Edge Intelligence, Real-Time Inference, and Lifecycle Maintenance

The fourth major issue concerns real-time deployment and operational maintenance. In sensor-enabled intelligent detection, inference is embedded in a larger loop that includes data acquisition, preprocessing, transmission, scheduling, actuation, and human review. Consequently, deployment feasibility cannot be judged only by parameter count or average inference time on a benchmark device. It must be evaluated in terms of end-to-end latency, throughput, memory usage, energy consumption, communication dependence, and runtime stability under field conditions. Recent reviews of edge intelligence and edge AI emphasize exactly this point: the practical challenge is not merely to compress a model, but to co-design architectures, compilers, hardware, and runtime policies so that the full sensing-to-inference pipeline remains efficient and dependable [23]. This is particularly important in machine maintenance, industrial inspection, and medical edge settings, where delays, jitter, or unstable connectivity may directly reduce system usefulness.

For this reason, resource-aware deployment techniques should be interpreted as systems-level necessities rather than optional optimizations. Model compression, quantization, pruning, operator fusion, and hardware–software co-design all matter because they determine whether intelligent detection can move closer to the sensing front end, reduce communication cost, and satisfy latency service-level objectives. Recent work on edge inference with dynamic architectures and network variability further shows that maintaining high percentile latency guarantees may require adaptive serving strategies rather than static deployment alone [107,108]. Application-oriented studies in predictive maintenance likewise demonstrate that edge AI solutions are attractive because they reduce latency and improve privacy, but they also make computational budgeting, thermal limits, update policies, and hardware compatibility central design concerns. Therefore, in a sensor-driven review, edge intelligence should be treated as part of the model evaluation problem, not merely as post hoc engineering.

Lifecycle maintenance is the final piece of this systems discussion. Even if a model is well calibrated and efficiently deployed at launch, its performance may degrade as sensor conditions, class distributions, operating regimes, or user behavior evolve. Recent work on concept drift detection and cost-aware retraining makes it clear that deployment should be treated as a continuous monitoring process rather than as a one-time release event. In practice, this means that sensor-driven intelligent detection systems require mechanisms for drift monitoring, trigger-based retraining, validation under new sensing conditions, rollback policies, and documentation of model updates [102,145]. Such lifecycle thinking is especially important in industrial and safety-sensitive settings, where deployment failure is often gradual rather than instantaneous. A mature sensor-enabled detection system is therefore not just a trained model but a maintained operational artifact with explicit monitoring, recalibration, and retraining policies. This is precisely why the old “training strategies” material is better absorbed into systems, evaluation, and governance than retained as a standalone algorithmic chapter.

## 8. Comparative Synthesis and Future Directions

The preceding sections reviewed intelligent detection from the perspectives of methodological evolution, sensor-driven problem formulation, and systems-level deployment constraints. A remaining task, however, is to move from description to synthesis. A review can only justify its analytical framework if it clarifies not merely what methods exist, but how different methodological families respond to different sensing challenges, where their strengths are robust, where they remain fragile, and which claims in the current literature are more ambitious than the available evidence warrants. This is particularly important in sensor-driven intelligent detection because performance is determined jointly by sensing conditions, representational choices, deployment context, and evaluation protocol. As a result, comparative synthesis should not be organized only around model names or benchmark rankings. It should instead compare methods against recurrent sensing problems, operational constraints, and the evidential standards required for reliable deployment. Recent work on real-world industrial anomaly benchmarks [37], uncertainty-aware medical AI, federated prognostics [135], and edge intelligence all point in this direction, namely that practical maturity depends on whether models remain valid under heterogeneous sensing, incomplete observations, domain shift, and lifecycle maintenance rather than on peak benchmark accuracy alone.

### 8.1. Cross-Domain Comparative Synthesis

A useful cross-domain synthesis must begin by making explicit what different method families actually solve. Table 2 summarizes the comparative relation between major methodological families and recurring sensor-driven challenges. The entries are not intended as absolute rankings. Rather, they express the dominant operational profile of each family under realistic sensing constraints. In particular, the comparison makes visible a structural asymmetry that recurs throughout this review. Traditional pipelines remain comparatively strong in mechanism prior and edge efficiency [23,30], deep learning substantially improves learned representations, foundation models improve representational breadth and semantic extensibility [5,6,77,78,81], while physics-informed methods strengthen mechanism consistency without automatically improving semantic transfer or multimodal interoperability [73]. Federated and privacy-preserving approaches, by contrast, contribute less at the level of raw representation quality than at the level of collaboration, governance, and cross-site robustness [101,135,146,147]. This helps explain why no single method family currently resolves all major sensing challenges simultaneously.

A second comparative dimension concerns the relationship between application domain, sensor modality, and evaluation risk, as shown in Table 3. Across domains, the dominant failure mode of a method is rarely determined by architecture alone. It is determined by the mismatch between what the sensing chain can reliably observe and what the evaluation protocol assumes. In industrial anomaly detection, the central risks lie in overly curated imaging conditions, weak representation of domain shift, and unrealistic false-positive tolerances. Recent datasets such as AeBAD, Real-IAD, and ISP-AD were proposed precisely because earlier benchmarks underrepresented domain variation, multiview inspection, weak-contrast defects, and factory-floor realism. In medical image analysis, the main evaluation risks concern acquisition bias, population heterogeneity, and overconfidence under variable image quality. In prognostics and health management, the main risks concern temporal leakage, unrealistic stationarity assumptions, and weak coverage of shifting operating regimes [113]. In process monitoring and soft sensing, evaluation may overstate validity if hidden-state observability, regime switching, or calibration quality are underrepresented. These patterns indicate that benchmark design is inseparable from sensing realism [141].

A third comparative dimension concerns the trade-off between model capability and deployment cost, as shown in Table 4. Much of the current literature reports gains in capability without making the corresponding system cost equally visible. Yet in sensor-driven intelligent detection, capability and cost must be judged together because deployment occurs inside latency-sensitive and often safety-sensitive workflows. CNN-based visual models improve localized perceptual sensitivity, but remain vulnerable to domain shift and image-quality variation [37]. Temporal deep models extend dynamic pattern extraction, but are still limited by asynchronous sensing, sequence length, and incomplete observations [148]. Generative models help with rare anomalies and sparse labels, yet often add inference cost and may not yield stable operational gains [149]. Foundation models improve transfer and semantic extensibility [6,77,150], but often incur substantial computational, documentation, and trustworthiness burdens [23]. Physics-informed methods improve feasibility and physical admissibility, but depend heavily on prior validity and may be difficult to optimize [73]. Federated collaboration improves governance and privacy compliance, but introduces communication overhead, aggregation instability, and cross-client heterogeneity management [135,140]. This comparative view makes it possible to evaluate methods not only by what they can do, but also by what they require and how they fail.

These three comparative views lead to a common conclusion. Intelligent detection has not evolved toward a single dominant solution. Instead, it has evolved toward a growing differentiation of methodological roles. Methods are becoming more specialized with respect to the particular sensing challenge they address, while the practical system increasingly depends on how such methods are combined, evaluated, and governed. This is why comparative synthesis is more informative than model listing. It reveals that apparent methodological progress is often uneven across sensing realism, deployment feasibility, and reliability under drift.

### 8.2. What Is Mature, What Is Fragile, What Is Overclaimed

A historically useful way to summarize the field is to reinterpret it as three successive paradigm changes. The first is the stage of explicit prior encoding, in which signal processing, handcrafted features, chemometrics, statistical process monitoring, and physically structured preprocessing dominated. In this stage, models were comparatively weak in representational flexibility but often strong in interpretability, low-resource deployment, and mechanism prior. The second is the stage of implicit representation learning, in which deep networks learned features end-to-end from raw or weakly processed sensor data. This stage greatly expanded the ability to model complex patterns in image, vibration, acoustic, and multimodal streams, but still remained heavily dependent on task-specific supervision and benchmark-bound optimization. The third is the stage of representation unification and knowledge-enhanced interaction, in which foundation models and multimodal language-linked systems introduced reusable latent spaces, open-vocabulary interfaces, and stronger alignment between sensor observations and text, prompts, or downstream reporting [5,6,77,78,81,101]. This three-stage interpretation clarifies that the current field is not simply accumulating models. It is repeatedly reorganizing the relation between prior knowledge, learned representation, and system-level usage.

Within this trajectory, some capabilities can now be considered comparatively mature. For image-centered defect detection, deep visual architectures and multiscale CNN-based detectors are mature in the sense that the dominant design principles are well established and performance under standard visual benchmarks is often high [7,37]. For classical fault diagnosis under controlled sensing regimes, temporal deep learning and hybrid preprocessing plus sequence modeling can also be considered mature at the level of methodology, even if not always at the level of cross-site generalization [20]. Multimodal fusion is mature as a conceptual necessity and as a taxonomy of data-level, feature-level, and decision-level integration [2]. Likewise, calibration, uncertainty quantification, and model documentation are mature as recognized requirements of trustworthy deployment, even though their actual implementation remains uneven. Edge-aware deployment strategies are also increasingly mature as a systems engineering agenda, because latency, compression, hardware–software co-design, and service constraints are now routine considerations in industrial AI [23].

Other capabilities remain fragile. Cross-domain validity remains fragile because many benchmarks still underrepresent device variation, environmental shift, and operational heterogeneity [113]. Missing-modality robustness remains fragile because many multimodal systems still assume full or near-full modality availability during design and evaluation [22]. Trustworthiness remains fragile because confidence quality, auditability, and failure documentation often lag behind representational performance. Federated collaboration remains fragile because privacy-preserving training does not eliminate client heterogeneity [140], and real distributed settings often contain significant sensing mismatch across participants [135]. Physics-informed and mechanism-constrained approaches remain fragile in a different sense, namely that their practical success depends on the availability, validity, and tractability of the embedded physical prior [73]. Foundation-model-based sensor intelligence is also fragile in deployment terms because its gains in semantic flexibility are often faster than its gains in calibration, resource efficiency, and controlled behavior under domain shift.

The most important category, however, is what is currently overclaimed. The literature sometimes overclaims that strong benchmark performance implies practical deployability, that multimodal fusion automatically guarantees complementary evidence use [2], that foundation models necessarily yield robust open-world sensor intelligence [5,6,77], or that privacy-preserving collaboration can be solved by federated optimization alone [135]. Real-world industrial benchmarks already show that many anomaly detection methods saturate on easier datasets but separate sharply on more realistic data [7,20,37]. Reviews of uncertainty quantification in medical image analysis and of model documentation likewise show that interpretability and calibration are not automatically improved by using larger or more general models. In the same way, federated prognostics literature increasingly makes clear that privacy and cross-domain adaptation are coupled rather than separable, while edge-AI studies show that model compression alone does not solve lifecycle maintenance or runtime instability [23]. These overclaims should not be dismissed as isolated rhetoric. They are structural signs that representational progress is currently outpacing evaluation realism and governance maturity.

For this reason, a more disciplined comparative reading of the field leads to a balanced judgment. Intelligent detection is genuinely progressing, but not uniformly. The most mature advances concern representation learning and multimodal integration under partially controlled conditions [2]. The most fragile areas concern transfer across heterogeneous sensing environments, reliable uncertainty behavior, and sustained deployment under operational change. The most overclaimed areas concern semantic generality, benchmark realism, and the ease with which collaboration and deployment can supposedly be solved by scaling up models. This is the point at which synthesis becomes more valuable than description, because it clarifies not only what the field has achieved, but also where its current self-understanding remains incomplete.

### 8.3. Future Directions for Sensor-Driven Intelligent Detection Systems

Building on the comparative synthesis above, future progress in sensor-driven intelligent detection is unlikely to be determined by model architecture or scaling alone. Instead, the next stage of development is more plausibly organized around five closely related directions that arise from persistent limitations in sensing realism, evaluation validity, collaborative learning, and deployment sustainability.

The first future direction is mechanism-aware modeling. The literature increasingly suggests that future progress in sensor-driven intelligent detection will not come solely from larger or more flexible models, but from better integration between sensing evidence and mechanistic structure. This includes physics-informed learning, operator learning, degradation-aware latent modeling, simulation–measurement fusion, and hybrid architectures that combine pretrained backbones with explicit process constraints [73,149]. The importance of this direction is strongest in prognostics, soft sensing, structural monitoring, and non-destructive evaluation, where latent-state inference must remain physically admissible rather than merely statistically plausible. A mature next step is therefore not only to improve predictive accuracy, but to make mechanistic regularization scalable, verifiable, and compatible with heterogeneous sensor inputs.

The second future direction is trustworthy evaluation and calibration. Future systems will need evaluation protocols that routinely integrate discrimination, calibration, uncertainty, abstention, and workflow relevance. This implies moving beyond accuracy-first reporting toward deployment-aware evidence standards. Confidence quality should be examined under domain shift, low-quality sensing, missing modalities, and rare-event settings [42]. At the same time, documentation practices should evolve from sparse model cards toward audit-oriented reporting that states sensing assumptions, domain coverage, validation protocol, and known failure modes. For sensor-driven intelligent detection, this direction is not merely an ethical add-on. It is a technical requirement for reliable operation in industrial and medical contexts.

The third future direction is promptable multimodal systems with missing-modality robustness. Foundation models and multimodal sensor intelligence have made clear that semantic conditioning, prompt-based adaptation, and shared latent spaces can improve extensibility [5,6,77,78,81]. Yet future progress depends on making these systems robust when modalities are incomplete, asynchronous, or quality-degraded [22]. Promptability should therefore be understood broadly, including text prompts, missing-signal prompts, task prompts, and adaptive multimodal routing under partial observability. In practical terms, the goal is not to maximize semantic breadth alone, but to make multimodal systems robust under the kinds of sensing incompleteness that real deployments routinely encounter [2]. This direction directly links representational unification with alignment and robustness, which is why it is central rather than optional.

The fourth future direction is privacy-preserving and cross-site collaboration. The next generation of sensor-enabled intelligent detection systems will increasingly depend on distributed collaboration across factories, institutions, and devices. This makes privacy-preserving learning, federated adaptation, and source-free transfer especially important [135,140,143]. However, the central research problem is not simply how to protect local data. It is how to preserve reliability under client heterogeneity, uneven sensing conditions, and incomplete domain overlap. Future progress therefore requires combining federated optimization with personalization, cross-domain adaptation, confidence-aware aggregation, and auditable collaboration protocols. In this setting, privacy and generalization should be treated as jointly optimized system properties rather than as separate goals.

The fifth future direction is edge-native and lifecycle-aware deployment [124,151]. Edge intelligence should evolve from a focus on lightweight inference toward a broader conception of sustained operational maintenance. This includes compression-aware model design, latency-sensitive serving, drift monitoring, trigger-based retraining, staged rollout, rollback procedures, and hardware–software co-optimization [23]. In real sensor systems, the decisive question is not whether a model can run once on an edge device, but whether it can remain reliable as data distributions, sensing quality, and operating conditions evolve. The concept-drift and edge-AI literature increasingly points toward this lifecycle view, in which deployment is treated as a monitored and adaptive process rather than as the terminal step of model development. This is especially important for industrial and safety-critical sensing, where operational drift is often gradual, persistent, and expensive to ignore.

Taken together, these five directions indicate that future progress in intelligent detection will depend less on a single dominant architecture [5,113,140] than on the ability to coordinate mechanisms, evaluation, collaboration, and deployment within heterogeneous sensing systems. In that sense, the future of intelligent detection is unlikely to be defined by a simple continuation of model scaling. It will be defined by whether broader representational capacity can be translated into sensor-grounded, trustworthy, collaborative, and maintainable intelligence under real operational conditions.

## 9. Conclusions

Intelligent detection should no longer be understood as a purely image-centered artificial intelligence task. Rather, it is more appropriately formulated as a sensor-driven state inference problem in which the observability, reliability, and operational value of model outputs are jointly determined by sensing physics, data quality, multimodal alignment, and system-level deployment conditions. From this perspective, the present review has argued that methodological progress in intelligent detection is inseparable from the structure of the sensing process itself, and that the field should therefore be interpreted through the coupled dimensions of methods, systems, and governance rather than through architecture-level innovation alone.

The comparative analysis developed in this review leads to several central conclusions. Traditional methods remain valuable in settings characterized by limited data, strong prior knowledge, and clear physical structure, where interpretability and low-cost deployment are critical. Deep learning substantially broadened the representational capacity of intelligent detection by enabling implicit feature learning from visual, temporal, and multimodal sensor data, while also extending into generative modeling and mechanism-constrained learning. Foundation-model-based approaches further strengthened representation unification, semantic extensibility, and cross-modal interaction, thereby creating new possibilities for reusable perceptual backbones and knowledge-enhanced reasoning. At the same time, these advances do not remove the fundamental constraints imposed by real sensing environments. Domain drift, missing modalities, calibration instability, privacy-preserving collaboration, and edge-side resource limits remain persistent barriers to reliable deployment, which means that apparent gains in representational generality do not automatically translate into operational trustworthiness.

Accordingly, the next stage of intelligent detection should not be judged primarily by isolated improvements in benchmark accuracy. Its real progress will depend on whether sensing, computation, decision support, and governance can be integrated into a reliable closed loop. In this sense, future sensor-driven intelligent detection systems must be not only more accurate, but also more physically grounded, more robust under heterogeneous sensing conditions, more transparent in uncertainty and calibration behavior, more compatible with privacy-preserving and collaborative learning, and more maintainable across the full deployment lifecycle. The decisive question is therefore no longer how to optimize a single detector in isolation, but how to build sensor-enabled intelligent systems whose performance remains dependable when measurements, environments, and operational demands continue to change.

## Figures and Tables

**Table 1:** Representative traditional methods in sensor-driven intelligent detection and their typical advantages and limitations.

Detection Type	Representative Method	Main Advantages	Main Limitations
Industrial defect detection	Magnetic particle inspection/penetrant testing	Low cost; simple operation	Applicable only to surface defects
Spectral Analysis	PCA + SVM	Strong interpretability	Highly dependent on feature engineering
Fault Diagnosis	Handcrafted Features + Random Forest	Strong noise resistance	Limited cross-scenario adaptability
Object Detection	HOG + SVM	Low computational complexity	Sensitive to background and illumination variations

**Table 2:** Method family versus recurrent sensor-driven challenges.

Method Family	Heterogeneity	Asynchrony	Missing Modality	Drift	Edge Deployment	Mechanism Prior
Handcrafted + shallow ML	weak	weak	weak	weak to medium	strong	strong
CNN-based visual deep learning	weak to medium	weak	weak	weak	medium	weak
Temporal deep learning/Transformer	medium	medium	weak	weak to medium	medium to low	weak
Physics-informed models	weak to medium	medium	weak	medium to strong	low	strong
Generative models	medium	weak	medium	weak	low to medium	weak
Foundation models	strong	medium	medium to strong	weak to medium	weak to medium	weak
Federated/privacy-preserving methods	medium	medium	weak	medium	medium	governance-oriented

**Table 3:** Application domain, dominant sensing modality, and major evaluation risks.

Application Domain	Dominant Modalities	Main Evaluation Risk
Industrial surface defect/anomaly detection	RGB image, multi-view image, thermal image	overly clean imaging conditions, weak domain-shift realism, inflated AUROC under easy settings
Fault diagnosis of rotating machinery	vibration, acoustic emission, thermal, current	operating-condition shift, sensor-position dependence, weak cross-site generalization
Remaining useful life/prognostics	telemetry, degradation trajectories, process variables, vibration	temporal leakage, unrealistic stationarity, weak evaluation under unseen degradation regimes
Medical lesion analysis/medical imaging	clinical image, dermoscopy, multimodal clinical text	acquisition bias, population shift, confidence miscalibration, workflow mismatch
Process monitoring/soft sensing	process variables, electrical signals, inferred latent states	hidden-state observability gaps, regime switching, calibration drift, indirect-label uncertainty
Structural health monitoring/NDT	strain, guided waves, ultrasound, thermal, imaging	environmental variability, sensitivity to excitation geometry, incomplete internal-state observability

**Table 4:** Model capability, dominant cost, and typical failure mode.

Method Family	Primary Capability	Main Cost	Typical Failure Mode
Handcrafted + shallow ML	interpretability, low-resource deployment, strong prior use	manual engineering burden	poor cross-domain transfer, brittle under heterogeneous sensing
CNN-based visual deep learning	strong local visual discrimination	annotation demand, dataset dependence	sensitivity to imaging shift and unseen defect morphology
Temporal deep learning/Transformer	dynamic correlation modeling over sensor streams	sequence complexity, data demand	instability under asynchronous or missing streams
Physics-informed models	physically admissible inference, structured extrapolation	prior specification, optimization stiffness	degradation when physics prior is incomplete or mis-specified
Generative models	anomaly modeling, scarce-label support, reconstruction-based screening	high inference cost, unstable operational gains	synthetic–real mismatch, unreliable localization
Foundation models	reusable backbones, semantic extensibility, transfer across tasks	compute, adaptation complexity, trustworthiness burden	hallucination, weak calibration, overclaimed generality
Federated/privacy-preserving methods	collaboration without raw-data sharing	communication cost, heterogeneity management	inconsistent convergence, uneven client performance

## Data Availability

No new experimental data were created in this study. The literature analyzed in this review was obtained from publicly accessible scholarly databases and published sources, as described in the manuscript. All data supporting the conclusions of this article are contained within the article and its reference list.
